# Bacterial Adherence and Dwelling Probability: Two Drivers of Early Alveolar Infection by *Streptococcus pneumoniae* Identified in Multi-Level Mathematical Modeling

**DOI:** 10.3389/fcimb.2018.00159

**Published:** 2018-05-15

**Authors:** Guido Santos, Xin Lai, Martin Eberhardt, Julio Vera

**Affiliations:** Laboratory of Systems Tumor Immunology, Department of Dermatology, Universitätsklinikum Erlangen and Faculty of Medicine, Friedrich-Alexander University Erlangen-Nürnberg, Erlangen, Germany

**Keywords:** *Streptococcus pneumoniae*, pneumonia, alveolar infection, biofilm, mathematical modeling

## Abstract

Pneumococcal infection is the most frequent cause of pneumonia, and one of the most prevalent diseases worldwide. The population groups at high risk of death from bacterial pneumonia are infants, elderly and immunosuppressed people. These groups are more vulnerable because they have immature or impaired immune systems, the efficacy of their response to vaccines is lower, and antibiotic treatment often does not take place until the inflammatory response triggered is already overwhelming. The immune response to bacterial lung infections involves dynamic interactions between several types of cells whose activation is driven by intracellular molecular networks. A feasible approach to the integration of knowledge and data linking tissue, cellular and intracellular events and the construction of hypotheses in this area is the use of mathematical modeling. For this paper, we used a multi-level computational model to analyse the role of cellular and molecular interactions during the first 10 h after alveolar invasion of *Streptococcus pneumoniae* bacteria. By “multi-level” we mean that we simulated the interplay between different temporal and spatial scales in a single computational model. In this instance, we included the intracellular scale of processes driving lung epithelial cell activation together with the scale of cell-to-cell interactions at the alveolar tissue. In our analysis, we combined systematic model simulations with logistic regression analysis and decision trees to find genotypic-phenotypic signatures that explain differences in bacteria strain infectivity. According to our simulations, pneumococci benefit from a high dwelling probability and a high proliferation rate during the first stages of infection. In addition to this, the model predicts that during the very early phases of infection the bacterial capsule could be an impediment to the establishment of the alveolar infection because it impairs bacterial colonization.

## Introduction

Pneumonia is a highly prevalent disease that kills almost 1 million children annually (McIntosh, 2002). The most frequent cause of pneumonia is infection with a strain of *Streptococcus pneumoniae* (*S.p*. or pneumococcus) (Sharma et al., 2007). Elderly and immunosuppressed people are high-risk groups because *S.p*. is an opportunistic pathogen (El-Solh et al., 2001; Wong and Evans, 2017). Although its virulence is limited, the pathogen achieves high morbidity because it is able to spread from permanent reservoirs in the nasopharyngeal airways which it colonizes asymptomatically in 15% of the general population and in about 40% of children (Tuomanen and Masure, 1997; Sjöström et al., 2006; de Lastours et al., 2016). Nasopharyngeal colonies are also more resistant to antibiotics compared to colonies in the lung (Perez et al., 2014), which makes controlling or eradicating the disease in the population a highly complex endeavor. Vaccination against *S.p*. is possible, but the efficacy of the vaccine is low in the risk groups (children, the elderly, and immunosuppressed people) due to the limited immune response triggered by the vaccine in these groups (Sjöström et al., 2006; Nguyen et al., 2017). Bacterial pneumonia begins with an infection of the alveolar cavities, but nasopharyngeal colonization precedes alveolar infection (Mandell, 2009). When the alveolar infection is recognized by the host, the immune system triggers an acute response culminating in substantial inflammation of the surrounding tissue. The inflammation itself compromises the alveolar gas exchange and potentially endangers the life of the patient (Henriques-Normark and Tuomanen, 2013). The disease can also worsen into septicaemia or bacterial encephalitis, increasing the patient's mortality risk (Henriques-Normark and Tuomanen, 2013; Iovino et al., 2016). Under these circumstances, optimum protection of high-risk patients would encompass preventing or impeding the initial alveolar infection, i.e., at the asymptomatic stage of the disease.

*Streptococcus pneumoniae* is able to generate an asymptomatic biofilm structure with high antibiotic resistance in the nasopharyngeal tissue (Simell et al., 2012; Perez et al., 2014). This structure is a prerequisite for a successful infection of the alveolar tissue (Simell et al., 2012). Nasopharyngeal co-infections, for instance by *Haemophilus influenzae*, can lead to immune stress on *S.p*. and thus trigger the release of bacteria from the biofilm to the lower airways, from where they can reach the alveoli and initiate infection (Chao et al., 2015). This stage likewise involves phenotypic transition of the bacteria from an unencapsulated, avirulent phenotype to a virulent one with a capsule (Simell et al., 2012; Weiser et al., 2015). It has been established that *S.p*. in the biofilm adopts a transparent appearance due to the lack of a capsule, which makes them more adherent but less invasive (Brueggemann et al., 2004; Kadioglu et al., 2008). Further, some studies have found that encapsulated bacteria are better able to colonize the alveolar surface and other tissues (Brueggemann et al., 2004; Hammerschmidt et al., 2005). Apart from the capsule (Jedrzejas, 2001), it has not yet been established which specific virulence factors permit *S.p*. to colonize the lower airways. Though gene expression differences between the biofilm and the virulent phenotype have been recorded (Lanie et al., 2007), the availability of these data has not led to an understanding of the early stages of alveolar infection.

When bacteria enter the alveolar lumen, they are exposed to a set of immunological barriers that prevent or impede infection. During initiation of the infection, the most relevant barriers are the tightly sealed epithelium, the alveolar lining fluid, and the alveolar macrophages (Sherman and Ganz, 1992). The epithelial cell layer with its tight junction-mediated intercellular adhesion acts as a physical barrier (Knight and Holgate, 2003). Furthermore, the epithelial cells are able to recognize the pathogens and release chemokines and cytokines that recruit and activate immune cells (Diamond et al., 2000). The primary role of the chemokines thus produced, such as MCP-1, is to attract resident alveolar macrophages to the site of infection (Deshmane et al., 2009), but in later stages other pro-inflammatory cytokines and chemokines recruit and activate neutrophils, monocytes, and leucocytes from the blood (Craig et al., 2009; Shi and Pamer, 2011; Hickey and Westhorpe, 2013). In the lining fluid, there are many factors that can inactivate pathogens, for instance complement proteins (Kadioglu and Andrew, 2004; Martin and Frevert, 2005). Also, the lining fluid acts as a means for the clearing of particles (including pathogens) from the alveolus, as the fluid is continuously produced and flows outwards toward the alveolar opening, dragging suspended bacteria along (Lindert et al., 2007). Finally, the macrophages serve as a mobile defense mechanism against the first stages of bacterial infection (Moldoveanu et al., 2008; Wilson et al., 2015). These cells follow and phagocytise bacteria, eliminating them before the infection becomes productive.

To recruit other immune cells, the resident cells in the alveoli produce pro-inflammatory cytokines, such as IL-8 and IL-1β, as well as chemokines (Standiford et al., 1990; Descamps et al., 2012). The phagocytic function of alveolar macrophages is then sequentially supplemented in later phases of the infection by other phagocytic cells, namely neutrophils and monocyte-derived macrophages (Mizgerd, 2002; Goto et al., 2004; Craig et al., 2009). Much later, the adaptive immune system controls the disease in a targeted manner.

The response to bacterial infection in the lung alveoli involves interactions between several types of immune and epithelial cells. The activation of key phenotypes in these cells is driven by intracellular molecular networks, each of which is distinctively activated in the course of the infection (Eberhardt et al., 2016; Cantone et al., 2017). Interestingly, this biological system contains multiple intracellular and paracrine positive and negative feedback loops that regulate the immune response (Hoffmann et al., 2002; Ashall et al., 2009). This type of regulatory loop can generate counter-intuitive, non-linear behavior (Tyson et al., 2003). Deriving hypotheses on the pathogenesis of lung infection that acknowledge these different levels of regulation, or integrating different types of experimental data accounting for the pathogenesis, is a difficult and onerous task even for a well-trained researcher.

A feasible approach to the integration of data linking tissue, cellular and intracellular events, the derivation of hypotheses and experiment design is the use of mathematical model simulations (Vera and Wolkenhauer, 2008; Cantone et al., 2017). This approach proceeds by collection of biomedical knowledge from publications and databases and its conversion into a graphical representation that connects the relevant processes, cells and molecules. On the basis of specific heuristic rules, this representation is encoded in a mathematical model that consists of equations, computational rules and other mathematical entities. Quantitative experimental data are used to characterize the mathematical model by assigning values to the parameters in the model equations in a process termed calibration. In a biomedical context, a calibrated model combined with experimental data can be used to dissect the fine tuning of regulatory pathways in immune cells or bacteria in the course of infection (Shih et al., 2012; Ben-Jacob et al., 2014), find new biomarkers for disease prognosis (Khan et al., 2017), analyse the feasibility of conventional or personalized treatments or detect new drug targets (Schoeberl et al., 2009; Passante et al., 2013), and gain understanding of complex ecological interactions between bacterial species during upper respiratory tract infection (Lysenko et al., 2010; Margolis et al., 2010; Mukherjee et al., 2014). In addition to this, models and their simulations can also be used to generate new hypotheses about the molecular and cellular pathophysiology of lung infection. Schultz et al. derived a mathematical model, calibrated with quantitative time series data, accounting for the interplay between macrophages and lung epithelial cells during *L. pneumophila* infection. They used simulation-based sensitivity analysis to conceive the hypothesis of a paracrine mechanism of macrophage-secreted IL-1β able to induce a prolonged degradation of the signaling factor IRAK-1 in lung epithelial cells. This model-based prediction was then corroborated with additional experiments (Schulz et al., 2017).

In line with this approach, we constructed, and characterized with available experimental data, a multi-level mathematical model derived to simulate the interactions between the host and *S.p*. inside a single alveolus during the first 10 h of infection. Previous work on multi-level modeling in the lung has sought to understand some key molecular interactions in fungal infections (Cilfone et al., 2013; Pollmächer and Figge, 2014, 2015; Oremland et al., 2016; Pollmächer et al., 2016). Our model combines ordinary differential equations (ODE) to simulate the activation of intracellular pathways in lung epithelial cells and an agent-based model to simulate the cell-to-cell interactions between bacteria, epithelial cells and macrophages. We confirmed that our mathematical model has predictive abilities by simulating the effect of removing some components of the model and comparing it with experimental observations. Next, we combined mathematical modeling-based simulations with data analysis techniques to derive phenotypic-genotypic signatures associated with bacterial infectivity. This methodology is an extension and adaptation of a previous workflow employed in the detection of cancer gene signatures (Vera et al., 2013; Santos et al., 2016; Khan et al., 2017). With our results, we constructed falsifiable hypotheses about the key molecular and cellular events in the early phase of lung *S.p*. infection and discuss them in the context of existing experimental evidence.

## Materials and methods

### Description of the multi-level mathematical model

**Figure 1:**
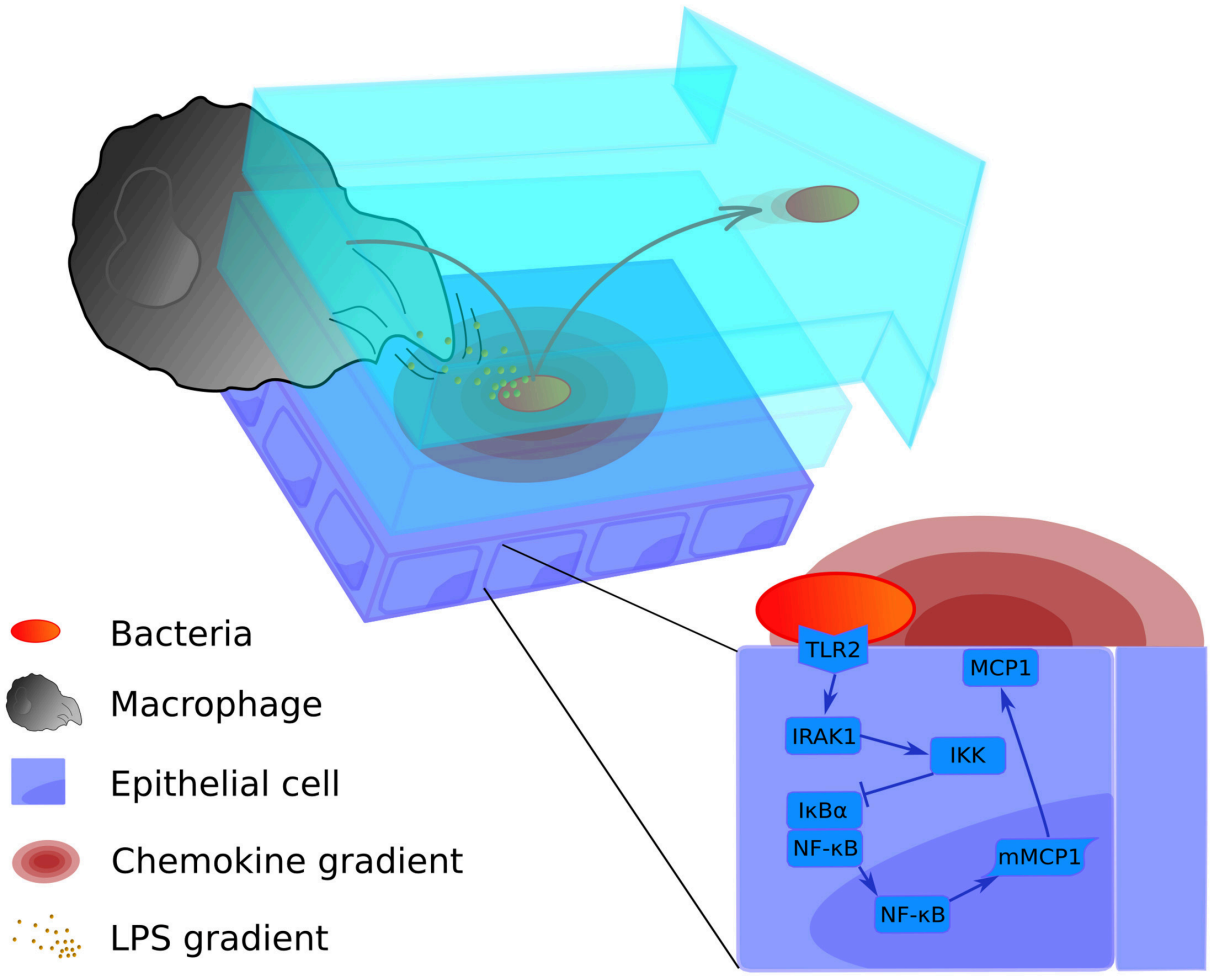
Graphical representation of the multi-level mathematical model of bacterial lung infection. The upper left panel displays processes at the tissue level of the model. It incorporates two lining fluid layers between which bacteria can transition (light blue layers). In both layers, the bacterial cell wall sheds factors (small orange particles) that attract nearby macrophages. In addition, when bacteria (red-orange oval) are attached to the epithelial cell layer (purple rectangles), they trigger the release of chemokine (pink concentric ovals) from the host epithelial cells; these chemokines act as a secondary attractive stimulus to macrophages (large gray structure). Bacteria can transition to the upper flowing layer (arrow-shaped blue layer), where they are moved by the flow toward the alveolar opening, and back again to the lower layer to re-attach at another site. The bottom right panel displays the intracellular level, containing the signaling pathway of the epithelial cells for production of the chemokine that attracts macrophages (MCP-1). For a more detailed scheme of the intracellular model, see Supplementary Material Figure S1.

Alveolar infection is a process that involves many elements at different scales in time and space. Our model traces the first 10 h of infection, in which the decision between abortive and productive infection is made. We have chosen 10 h for the simulations based on cytokine profile data from cultured lung epithelial cells activated by *S.p*. (Schmeck et al., 2006), which indicates a low concentration of cytokines before this time. After 10 h, the production of cytokines such as IL-1β increases, which modifies the activation profile of epithelial cells and alveolar macrophages (Schmeck et al., 2006). The model was implemented in MATLAB and the source code is available online at www.jveralab.net/resources. We define the nominal parametrization of our model as the situation of a healthy host quickly eliminating an initial infection of the alveolar tissue (see Results section and Table S1). The model simulates a single alveolus composed of 121 equally-sized epithelial cells (30 μm side length). We consider three interconnected modeling domains: (1) the lining fluid dynamics of the alveolus and bacterial proliferation, (2) the movement of macrophages and bacteria, and (3) the intracellular signaling inside epithelial cells of the alveolus (Figure 1). A detailed mathematical description of the model is included in Supplementary Material (*Intracellular signaling pathway* and *Tissue level scale of the model* sections).

The two levels of the model are juxtaposed in Figure 1: the intracellular level to the lower right and the tissue level to the upper left. We have described in Supplementary Material how the scales are combined in our model using an alternating simulation strategy (*Tissue and cellular scales merging and* modeling section).

#### Alveolar lining fluid and bacterial growth

The inner alveolar surface is modeled as a two-dimensional square-shaped landscape with two layers of lining fluid on top (see Figure 1). The lower layer is stationary while the upper layer flows continuously. The model assumes that the liquid is produced at a constant rate at the center of the landscape and flows radially toward the borders and finally out of the alveolus. The velocity of the flow is calculated from *in vivo* measurements (Lindert et al., 2007) (see Figure 1, upper panel, and Table S1). As *S.p*. is an extracellular pathogen, it proliferates and moves in the lining liquid on the alveolar surface. The movement follows a memoryless random walk model. The doubling time for *S.p*. is 200 min (Jakubovics and Palmer, 2013) (see Supplementary Material, Table S1), and the divisions occur asynchronously.

**Figure 2:**
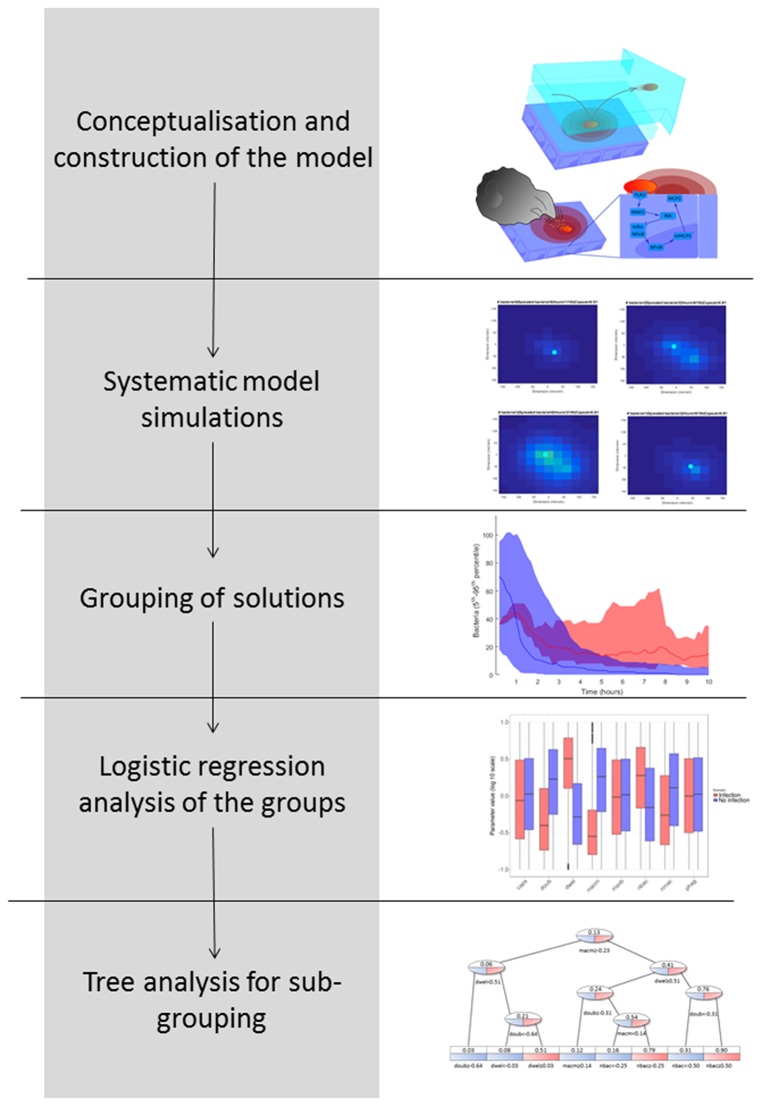
Workflow of the study.

There is a certain probability of bacteria passing between the two fluid layers in each time step. This probability value was optimized in order to produce balanced solutions in the nominal parametrization, such that bacteria neither immediately pass out of the alveolus nor remain permanently at one site (see Table S1). Bacteria in the stationary layer stay attached to the epithelial cells and move slowly, but when they are in the upper layer, their random walk is faster and they are also dragged along by the flow (see Supplementary Material, Table S1, sessile vs. floating). Additionally, bacteria trigger the production of MCP-1 in epithelia only when they are in the lower layer (Figure 1). Bacteria in the flowing layer can be dragged outside the modeled area; they are then supposed to have been cleared and are removed from the simulated alveolus, counting as “spread bacteria” (see Figure 2). These bacteria stop playing a role in the infection processes in the alveolus of interest, but we acknowledge that spread bacteria can enter other alveoli. We will consider this effect in relation to later infection stages in future improvements of the model.

#### Alveolar macrophages

Resident macrophages perpetually patrol the alveolar lumen to protect the lung from environmental agents and pathogen infection (Marriott and Dockrell, 2007). It can be estimated that in a single alveolus in physiological conditions, we can find between one and five alveolar macrophages (Wallace et al., 1992; Ochs et al., 2004) (see Supplementary Material, Table S1). Macrophages are not affected by the liquid flow because it is assumed that they move autonomously using pseudopodia attached to the epithelial layer. They have an average diameter of 21 μm (Krombach et al., 1997). The model assumes that unstimulated macrophages follow a random-walk movement pattern if they are not attracted by any gradient. Once the bacteria come into close contact with a macrophage, they remain attached, and are subsequently removed from the simulation (phagocytised). We use an exponential decay function to model the removal of attached bacteria through phagocytosis, which is consistent with experimental measurements of the quantification of bacterial phagocytosis *in vitro* (Athamna and Ofek, 1988) (see Supplementary Material, Table S1). If the number of attached bacteria on a macrophage surpasses a defined threshold for 1 min or more (see Supplementary Material, Table S1), the macrophage dies by apoptosis (Bewley et al., 2014). Apoptosis of macrophages promotes bacterial clearance (Aberdein et al., 2013). Initially, this threshold is set at 50 in accordance with the cellular surface ratio of the bacteria to the macrophage (see Supplementary Material, Table S1). However, our simulations analyse this parameter at a wide range of possible values to study its effect (see section *Model simulations*).

Macrophages are attracted to bacteria through two different gradients: a long-distance gradient defined by the MCP-1 chemokine produced by activated epithelial cells, and a short-distance gradient defined by factors such as peptidoglycans and lipopolysaccharides released from the bacterial cell wall (Dauber and Daniele, 1978; Dohlman and Goetzl, 1978; Fisher et al., 1988) (see Figure 1, lower panel, at left). This part of the model is as yet the least well-supported, because the role of chemoattractant signals has not been established at the small scale of a single alveolus. Nevertheless, previous *in silico* studies have utilized similar local gradients (Charnick et al., 1991; Pollmächer and Figge, 2014). In order to decide whether to include the chemoattractant signal in the model, we generated a set of 50 parameter configurations with random perturbations of the nominal parametrization within a 10% margin, and simulated each with and without the chemoattractant signal, respectively. We observed that only 1 (2%) of the solutions with the chemoattractant signal, but 17 (34%) of the solutions without the chemoattractant signal have more than 40 bacteria after 10 h of simulation. We therefore decided to include the local chemoattractant signal in our model in order to align the nominal parametrization with the predicted phenotypic response of a healthy host.

In our model, macrophages detect the chemoattractant signal of MCP-1 with an arbitrarily chosen threshold of 10^−6^ mM (see Supplementary Material, Table S1). This value is relatively low, but simulations show that it is not relevant to the results when modified across several orders of magnitude (data not shown). If a macrophage is as close to a bacterium as the length of one epithelial cell (see Supplementary Material, Table S1), this macrophage can detect the gradient of bacterial factors (Fisher et al., 1988) and move directly toward the pathogen. In this, we are assuming that, even when macrophages are exposed to bacterial peptidoglycans and lipopolysaccharides, they will rely only on the chemokine gradient for closing long-range distances.

#### Epithelial cell level and chemokines

For the intracellular part of the model, we have adapted an ODE model developed previously in our group (Schulz et al., 2017). In the original model, epithelial cells respond to the detection of *Legionella* via an NF-κB-mediated intracellular network. For the present study, we assumed that the intracellular network mediating NF-κB activation has the same structure, but we substituted the original receptor that recognizes the bacteria and the chemoattractant with TLR2 and MCP-1, respectively (TLR2) (see Supplementary Material) (Deshmane et al., 2009). The TLR2 receptor recognizes bacterial lipoproteins and phosphorylates IRAK1. The signal is released through activation of IKK, that phosphorylates and promotes the degradation of the NF-κB inhibitor IκBα (Koedel et al., 2003; Malley et al., 2003). When the inhibitor is eliminated, NF-κB is imported into the nucleus and activates the transcription of many pro-inflammatory genes, including chemokines such as MCP-1 (Shyy et al., 1993; Wang et al., 2000; Kim et al., 2006). MCP-1 is released from the cell to the lining fluid and produces a gradient that attracts the alveolar macrophages (Deshmane et al., 2009). The detailed description of the intracellular model is available in the Supplementary Material (*Intracellular signaling pathway* section). Each of the 121 epithelial cells runs its own instance of the ODE model and is able to individually respond to bacteria by producing and secreting MCP-1. The secreted MCP-1 diffuses through the epithelial cell layer following Fick's Law, generating a gradient that the macrophages can detect. It is assumed that MCP-1 diffuses through the lower laminar layer of the lining fluid. For a detailed explanation of the equations and parameters, see the *Intracellular signaling pathway* section in Supplementary Material.

### Computational workflow

In Figure 2, we explain the workflow we followed to obtain the results, pursuing a strategy adapted from previous studies (Vera et al., 2013; Santos et al., 2016). The aim of this workflow is to group simulation results according to biological phenotypes and by so doing to identify the influence of the model parameters in these phenotypes. The workflow proceeds as follows: (1) Conceptualization and construction of the mathematical model based on current knowledge; (2) Execution of systematic simulations in which the values of key model parameters are perturbed; (3) Grouping of the solutions based on the phenotypic response investigated; in this case, this was the predicted number of bacteria at the end of the model simulation; (4) Logistic regression analysis of the phenotypic groups in order to identify key model parameter differences; and (5) Decision tree analysis in order to identify phenotypic subgroups characterized by a distinctive set of key parameter values.

### Model simulations

The nominal parametrization of the model is defined as the closest reproduction of a physiological situation where *S.p*. attempts to colonize the alveolar tissue of a healthy adult. To obtain this parametrization, we assumed reasonable parameter values for the model if we were unable to derive them from the literature. In the Supplementary Material, Table S1 lists the parameters and the scientific papers from which their values were obtained. We focused on parameters that correspond to factors with high variability in the epidemiology of pneumonia. Different strains of *S.p*. display differences in bacterial doubling time (*doub*), in capsule production (*caps*) and in the presence of adherence receptors that influence the transition between the dynamic and the stationary layer of the lining fluid (*dwel*). The initial number of bacteria can also be highly variable (*nbac*) (Brueggemann et al., 2004; Serrano et al., 2006; Shen et al., 2006). To account for differences in immunological conditions in the hosts, we consider differences in the number of alveolar macrophages (*nmac*), and the macrophages' state of activation is reflected by their phagocytic activity (*phag*) and their mobility (*macm*) (Saito et al., 2008; Descamps et al., 2012). Finally, to account for the differing propensities of macrophages to undergo apoptosis, the number of bacteria that will trigger apoptosis in macrophages if ingested simultaneously is modified (*maxb*). The remaining parameters at the tissue level are assumed to remain constant between different hosts and pathogens. The intracellular parameters selected account either for mutations that affect protein or mRNA stability (*kirak1deg, kirak1pdeg, kikkdeg, kikkadeg, kmikbadeg, kikbaloss, kmmcp1deg*, and *kmcp1deg*) (Medvedev et al., 2003; Rose et al., 2003) or for mutations that affect the activation of proteins (*kirak1ph, kikkact*, and *knfkbgain*) (Boyd et al., 2012). To investigate the model, we first defined an accessible range around the nominal value for each of the selected parameters. We then obtained a high number of uniformly random parametrizations within these constraints and ran a simulation for each, and finally performed regression analysis of the phenotypic outcome on the basis of the parameter values.

We analyzed the parameters for the tissue level and the intracellular level independently, as the timescales for each are very different. For each analysis, we obtained a set of 2·10^4^ solutions. As the model contains stochastic parts, we repeated each run 10 times to obtain a measurement of the mean and standard error of bacteria numbers at the end of the simulation (10 h). We calculated the 95% confidence interval (CI) of the number of bacteria based on the mean and standard error for each solution. This confidence interval is used to group solutions such that a solution belongs to the group *high load* if the lower boundary of the CI is above 10 and to *low load* if its upper boundary is below or equal to 10.

All simulations were performed on a Dell PowerEdge R820 with 2 Intel Xeon E5-4650L CPUs and 128 GB main memory, running Ubuntu 14.04.5 and MATLAB R2015b. A single simulation took around 1 min in our setup.

### Logistic regression

In our analysis, we compare *high load* to *low load*. Logistic regression is advantageous in our situation because it accommodates binary-valued outcomes (Lever et al., 2016). The regression casts the model parameters as independent variables and the binary distinction as the dependent variable. The regression model is as follows:

$$\begin{array}{l} logit\left(p\right)=\ln\left(\frac{p}{1-p}\right)=\beta_{1}x_{1}+\cdots +\beta_{n}x_{n}, \end{array}$$

where p is the probability of belonging to one of the defined groups, which is transformed through the logit function into a linear continuous variable between –∞ and +∞ that can be predicted as a linear combination of the model parameters (x_n_). The factors β_n_ measure the influence of each parameter on bacteria numbers. They can be positive or negative depending on their correlation to the outcome. The absolute values of the factors β_n_ are considered in tests of significant differences between groups of simulations.

The logistic regression analysis was performed using the package *glm* in R (version 3.4.0).

### Decision tree analysis

Logistic regression analysis is only able to calculate the influence of each model parameter on the outcome of a binary variable (e.g., *high load* vs. *low load*), but it cannot differentiate subpopulations of solutions within *one* group of solutions. The identification of subpopulations, however, would be particularly interesting for our purposes because different strains of pneumococci (represented by different model parametrizations) may achieve successful infection using different strategies. To address this question, we employed a decision tree analysis using the package *rpart* in R (version 3.4.0). Trees are mathematical objects that can separate multidimensional objects into disjoint sets based on the separation of *one* dependent binomial variable. In our case, the multidimensional objects are the parameter configurations, and the dependent binomial variable to be separated is the bacterial load (0 for low load and 1 for high load). The sets defined by the tree are generated in such a way that the average values of the bacterial load inside a single set present the greatest differences while using the minimum amount of sets. Each new set is generated by a partition of *one* of the parameters into *two* parts (higher and lower than a defined value). The leaves of the tree represent the sets obtained, which in our case are interpreted as different strategies that the pneumococci might follow in order to infect the alveolus (decision tree, see Figure 2).

## Results

### Nominal model parametrization and validation

The model was tuned in such a way that, in the nominal parametrization, an infection with an everyday number of bacteria under the surveillance of a single resident alveolar macrophage leads to simulations that consistently finish at 10 h with a lower than initial bacterial load, here considered indicative of successful immune control. An everyday number of bacteria would correspond to a value lower than the minimum infectious dose for *S.p*. This infectious dose in experiments on mice is between 100 and 1,000 bacteria per alveolus (Orihuela et al., 2003). The nominal solution considers a realistic situation in a healthy individual in which frequent encounters with low numbers of *S.p*. would not produce alveolar infection, for which higher doses coming from nasopharyngeal colonization would be required (Mandell, 2009). To increase confidence in the nominal parametrization, we ran 100 simulations with the initial number of bacteria (25) following a normal distribution of bacteria concentration, with the mean at the center of the simulated alveolus. The center of the alveolus corresponds to the point opposite the alveolar entry, so the bacteria would accumulate around this point when they invade the alveolus from the entry. We observed complete clearance in 19 cases. The confidence interval for the number of bacteria at the end of the simulation is [7.9, 11.4] (CI 95%), and its upper boundary is less than half the initial number of bacteria.

**Figure 3:**
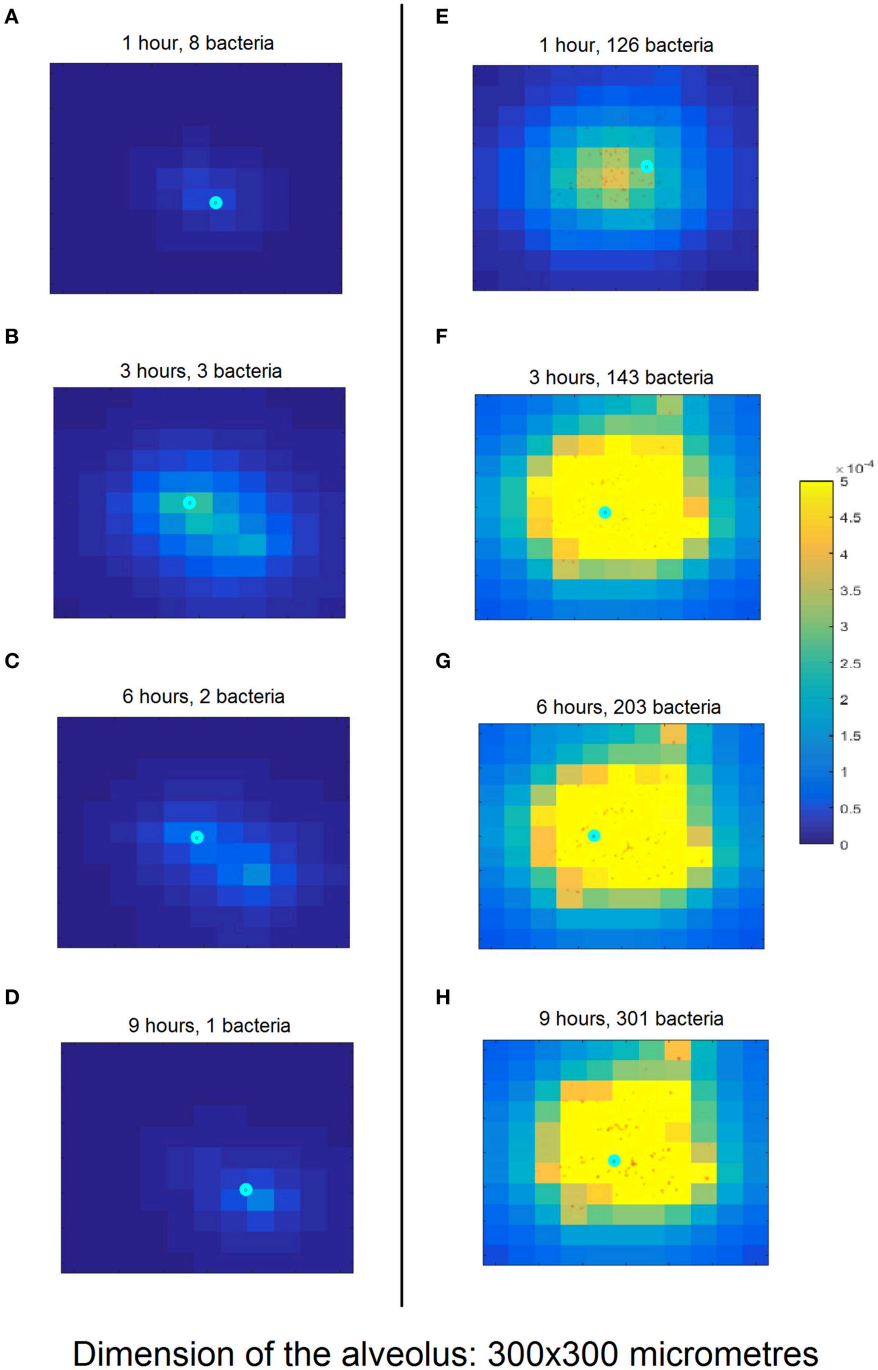
Snapshots of simulation progression at 1, 3, 6, and 9 h (cf. rows from top to bottom) for the nominal model parametrization **(A–D)** compared to an exemplary successful infection **(E–H)**. **(A)** The simulation was initiated with 10 bacteria (red dots) and one macrophage (cyan circle). The lighter background color around the bacteria represents the incipient production of MCP-1 from the epithelial cells (square domains in background) in this area. **(B)** The proliferating bacteria stimulate an increased release of MCP-1 and the macrophage follows the gradient. **(C)** The macrophage starts phagocytising the bacteria; the production of MCP-1 decreases. **(D)** The macrophage finds the last remaining bacteria and clears the infection. **(E)** The simulation begins with 100 bacteria which almost immediately trigger a strong chemokine release. **(F)** After 3 h and massive accumulation of MCP-1, the macrophage clears many bacteria. **(G)** The number of bacteria keeps increasing, dispersing across the alveolus. **(H)** After 10 h, the bacteria have spread through a wide area on the alveolus. The macrophage is not fast enough to clear all of them and the infection has become productive. Figure S3 provides a clearer representation of the small details of **(H)**.

The left-hand column in Figure 3 shows a simulation of the nominal scenario at 1, 3, 6, and 9 h after initiation of bacterial infection, while the right-hand column is an instance of a simulation with successful bacterial infection proceeding from a higher number of bacteria (150 bacteria). By contrast, in the nominal scenario the bacteria are cleared by the macrophage in <6 h.

To confirm the model's predictive abilities, we ran simulations in which we removed the ability of macrophages to follow the MCP1 chemo-attractant gradient and subsequently estimated bacterial population size at the end of the simulation with and without the MCP1 chemo-attraction (50 simulations each). We found that our model simulations are in accordance with recently published experimental data for wild-type and CCL2 knockout mice infected with *S. pneumoniae* (Winter et al., 2009) (see Figure S2).

### Tissue-level processes affecting infection progression

**Figure 4:**
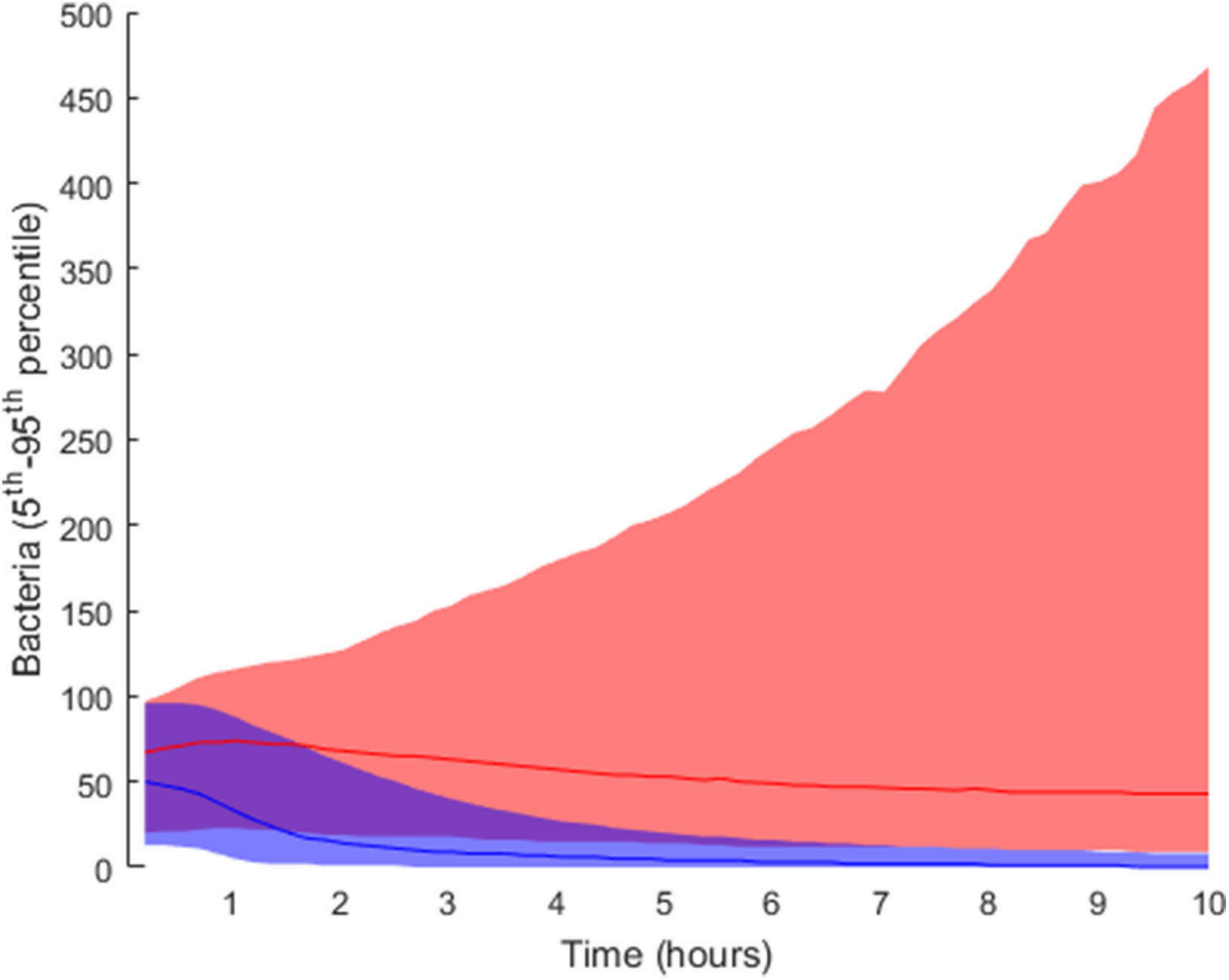
Distribution of final bacteria numbers in the simulations accounting for the influence of tissue level parameters. The figure shows the evolution over time of the interpercentile range between the 5th and 95th percentiles in the two groups: *high load* group in pink and *low load* in blue. The solid lines in darker shades represent the median of each group.

In order to elucidate the tissue-level processes that may affect the infection, we first selected a subset of biologically relevant parameters at the tissue level (see section Materials and Methods) and randomly perturbed them while keeping all the other model parameters at their nominal values. The simulations were then run accordingly and the results classified into groups. As described in the Materials and Methods section, we performed 2·10^4^ simulations by perturbing the relevant parameter values following a uniform distribution inside the intervals in Table 1). We ran 10 simulations for each solution to account for stochasticity in the simulations. The solutions were classified into two groups according to the lower boundary of the confidence interval for the number of bacteria at the end of the simulation: *high load* contained values higher than 10 bacteria (5,853 solutions), while the remaining solutions were classified as *low load* (14,174 solutions). The outcomes for both groups are shown in Figure 4, where the *high load* group (red) shows higher numbers of bacteria toward the end than the *low load* group (blue).

**Table 1:** Tissue-level parameter search space.

**Parameter**	**Multiplier**
nbac: initial number of bacteria	[10,100] times
nmac: initial number of macrophages	[1,5] times
difk: diffusion constant of chemokines	[0.1,10] times
antg: number of antigens recognized per bacterium	[0.1,10] times
srfm: movement speed of bacteria on the surface	[0.1,10] times
flom: movement of bacteria in lining fluid	[0.1,10] times
radm: movement of lining fluid	[0.1,10] times
doub: doubling time of bacteria	[0.1,10] times
dwel: probability that bacteria will remain in the same phase	[0.991,1.001] times
satr: saturation of bacterial growth	[0.1,10] times
caps: kinetic constant for capsule production	[0,2] times
macm: macrophage movement speed	[0.1,10] times
phag: phagocytosis rate	[0.1,10] times
maxb: maximum bacteria per phagocytosis	[0.1,2] times
lpss: LPS sensitivity of macrophages	[0.1,10] times
cytg: MCP-1 sensitivity of macrophages	[0.1,10] times
All intracellular parameters (for details see Supp. Mat.)	[0.5,2] times

*The range is generated around the nominal value (see Supplementary Material, Table S1), with the exception of the number of bacteria and macrophages (nbac and nmac), and is represented as the factors by which the nominal value is multiplied. Note that the explanation for the intracellular-level parameters is given in Supplementary Material*.

**Figure 5:**
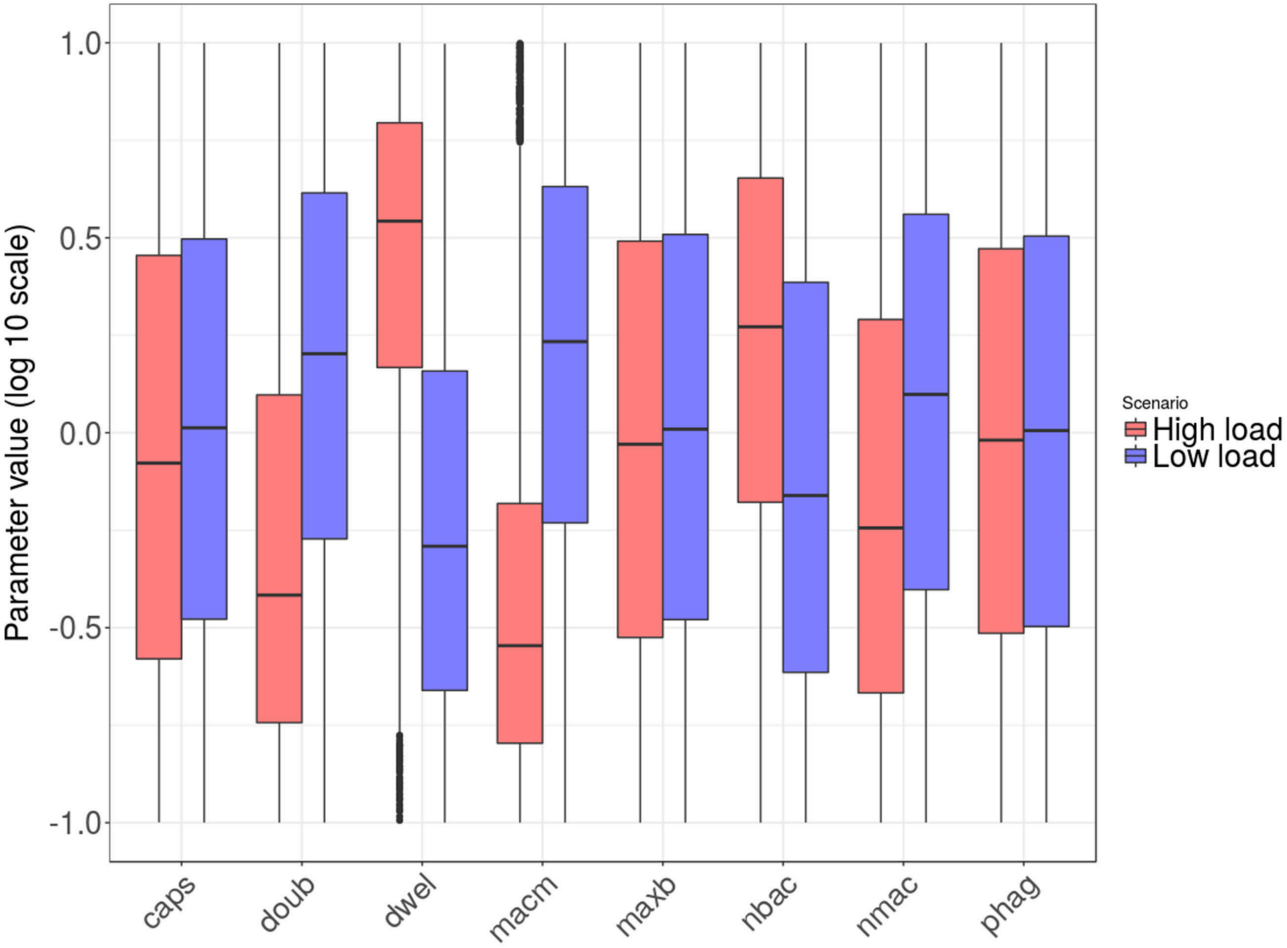
Box plots of tissue-level parameter value distributions in the two groups: *high load* group in red and *low load* in blue. Only the parameters revealing significant differences according to the logistic regression analysis are shown.

Figure 5 shows the distribution of the parameter values after transformation to logarithmic scale and linear projection from their original range to the interval [0.1, 10]. The parameters *nbac* and *nmac* were normalized without logarithmic transformation, as their factor range was linear.

Next, we wanted to identify the parameters with significant differences between the high and low bacterial load groups. To this end, we applied logistic regression, a statistical method specifically suited to accommodating binary-valued outcomes like those investigated here (see details in section Materials and Methods). The results indicate that six of the tissue-level model parameters distinguish the two groups. They can be aggregated as follows: (i) parameters related to bacterial phenotype (*nbac, doub, dwel*, and *caps*) and (ii) parameters related to macrophage phenotypes (*nmac* and *macm*). Regarding the first group, the parameter *dwel* is a measure of bacterial adherence that represents the dwelling probability, i.e., the probability that the bacteria will stay attached in the lower fluid layer instead of free-floating in the upper fluid layer until the next iteration. The values of *dwel* are higher in the *high load* group (red) than in the *low load* group (blue).

Moreover, the parameter accounting for bacterial doubling time (*doub*) is lower in the *high load* group, a result that was expected under the assumption that highly proliferative bacteria increase the chances of successful infection. The parameter *nbac* determines the initial number of bacteria in the simulation. These bacteria are randomly distributed through the alveolus following a normal distribution centered in the middle of the alveolus, and all of them are located in the upper layer of the lining fluid at the beginning of the simulation. The logistic analysis shows, unsurprisingly, a higher number of initial bacteria (*nbac*) in the *high load* group.

**Figure 6:**
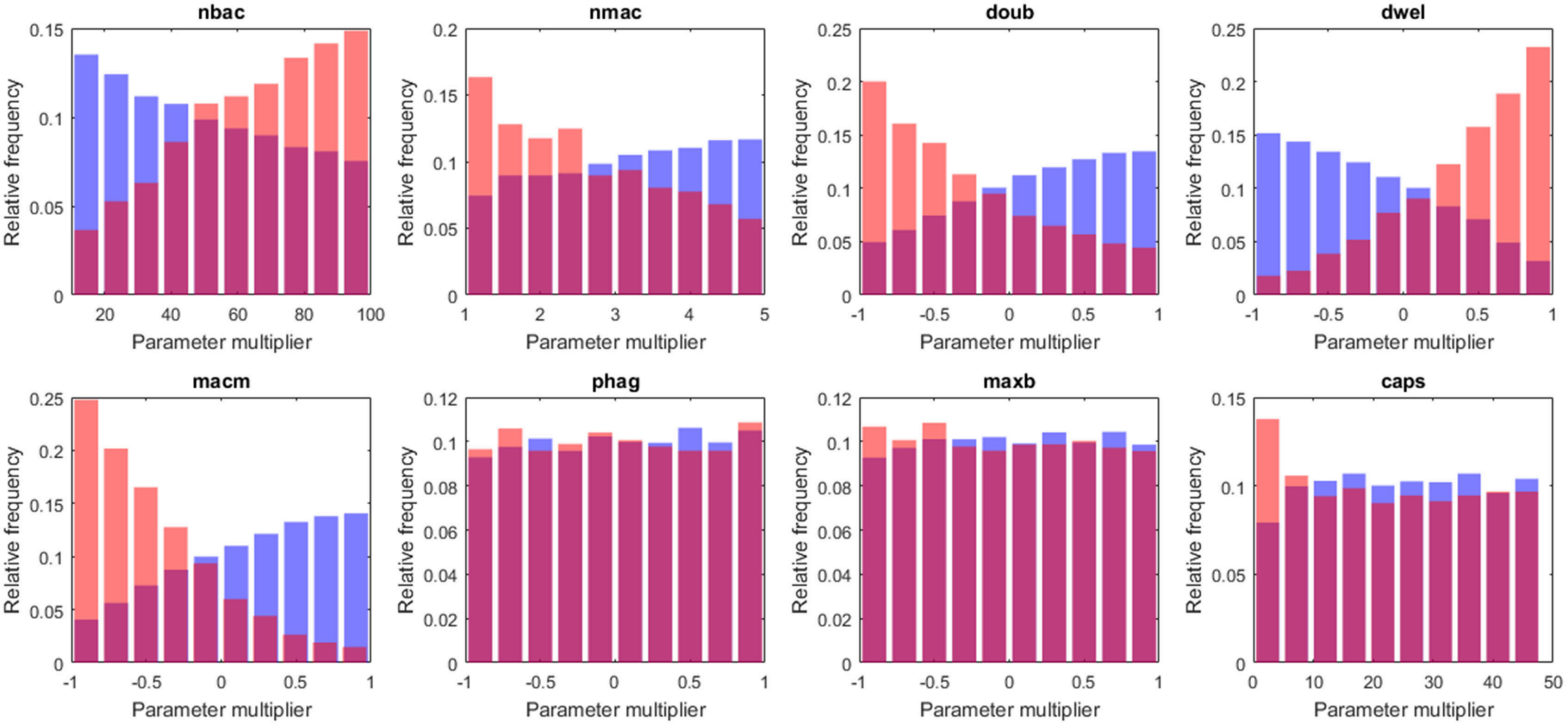
Histograms of tissue-level parameter values: *high load* group in red and *low load* in blue. The plotted values are the factors that multiply the value of the nominal solution. The distribution of solutions for a different sample number is shown in Supplementary Material.

The capsule represents the most important virulence factor for the infectivity of *S.p*. In the model, capsule production follows a saturation dynamic, and the parameter *caps* represents the velocity of the bacteria in capsule production (*caps* is the time taken to reach half the maximum capsule thickness). It has been observed that differences in capsule thickness between different strains arise from differences in the energy cost of capsule monomer production (Weinberger et al., 2009). The model reflects this specificity by tuning the parameter accounting for the rate of capsule production. The analysis of the results indicates differences between the populations of *high load* and *low load* solutions for low values of *caps* (Figure 6). The capsule prevents phagocytosis by the alveolar macrophages (AlonsoDeVelasco et al., 1995), which is an indication of why the model predicts an important role for capsule production in facilitating the infection. However, the capsule also decreases bacterial adherence and hence exposes the bacterium to the drag of the lining fluid (Adamou et al., 1998). We modified the model to account for this effect. The probability of transition from the flowing layer to the stationary layer of the lining liquid decreases with higher amounts of capsule. Specifically, if the amount of capsule is at its maximum, the bacteria will not settle, and if the amount of capsule is zero, the probability of settling will be at a maximum (but different from 1). The logistic regression analysis was then repeated with 2·10^4^ more solutions, with 10 repetitions each obtained with this new version of the model. When the trade-off between protection against macrophages and removal by the lining fluid was considered, the impact of the capsule production rate on infection is reduced by more than half (see Table S5).

According to our analysis, the decision on early bacterial clearance is influenced by phenotypic features of the macrophage, namely the number of macrophages and the movement of macrophages (*nmac* and *macm*, respectively) (see Figure 6). These two parameters attain higher values in the *low load* group than in the *high load* group. Other macrophage-related parameters, such as *maxb* (apoptosis propensity) and *phag* (phagocytosis rate), did not display differences between groups. The model predicts that during the early stages of infection, the leading physiological parameters of macrophages are their number and their velocity, and the phagocytosis rate and propensity to apoptosis do not play an important role.

### Intracellular processes affecting infection resolution

**Figure 7:**
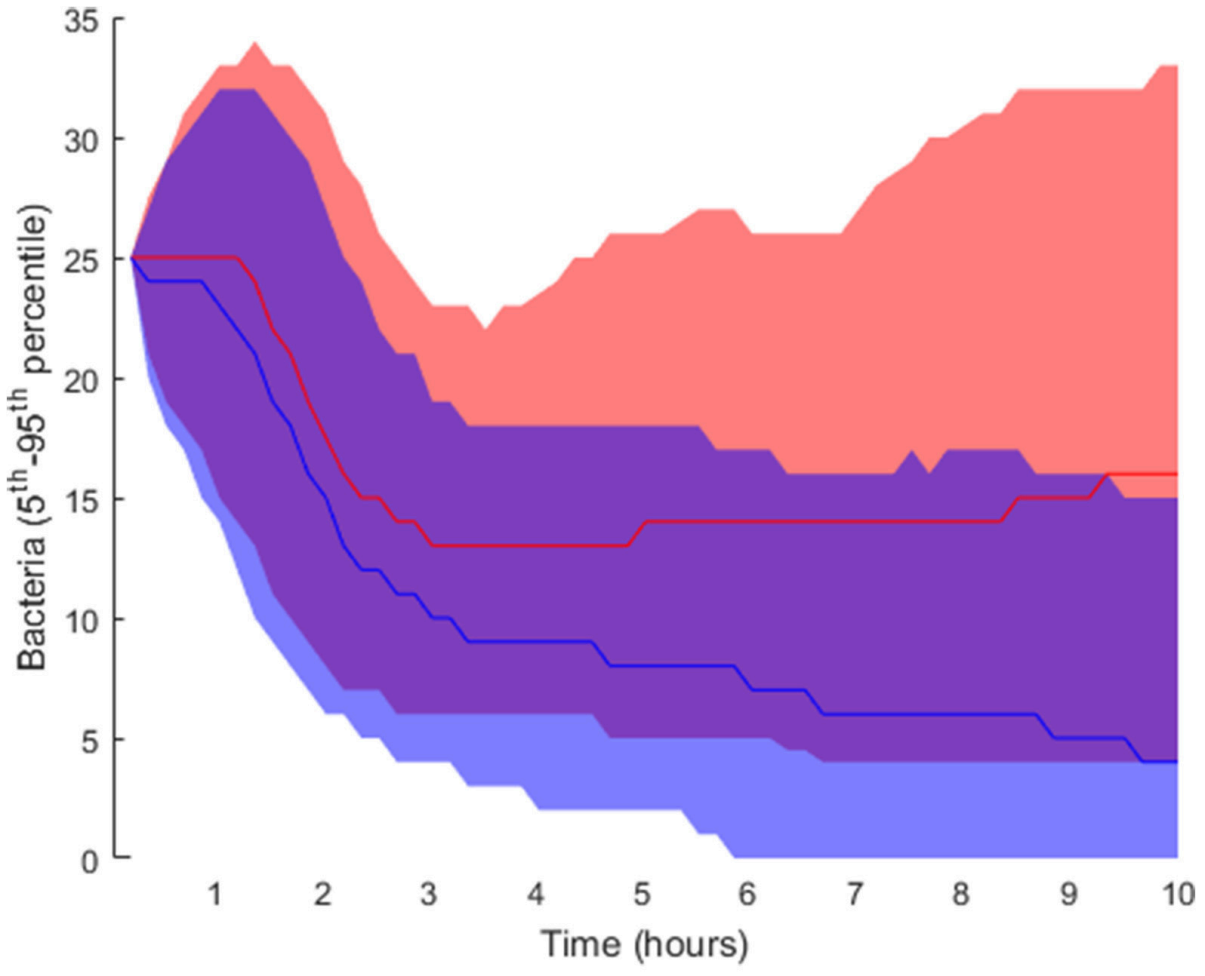
Interpercentile range between the 5th and 95th percentiles for final bacteria numbers of the intracellular-level solutions for the *high load* group in red and *low load* (blue). The lines represent the median of each distribution.

Regarding the intracellular level of the model, which accounts for the regulatory pathway that activates lung epithelial cells, the selected parameters were also randomly perturbed from their nominal values while all the other parameters were kept at their nominal values. In this way, we generated 2·10^4^ parameter sets used to perform simulations and classified them into groups (Table 1; further details in section Materials and Methods). We applied the same criteria for grouping the solutions as in the case of tissue-level parameters. Six thousand one hundred and nine solutions were grouped as *high load* and 15,448 solutions as *low load*. The 5th and 95th percentiles of bacteria numbers in the solutions thus grouped are shown in Figure 7.

**Figure 8:**
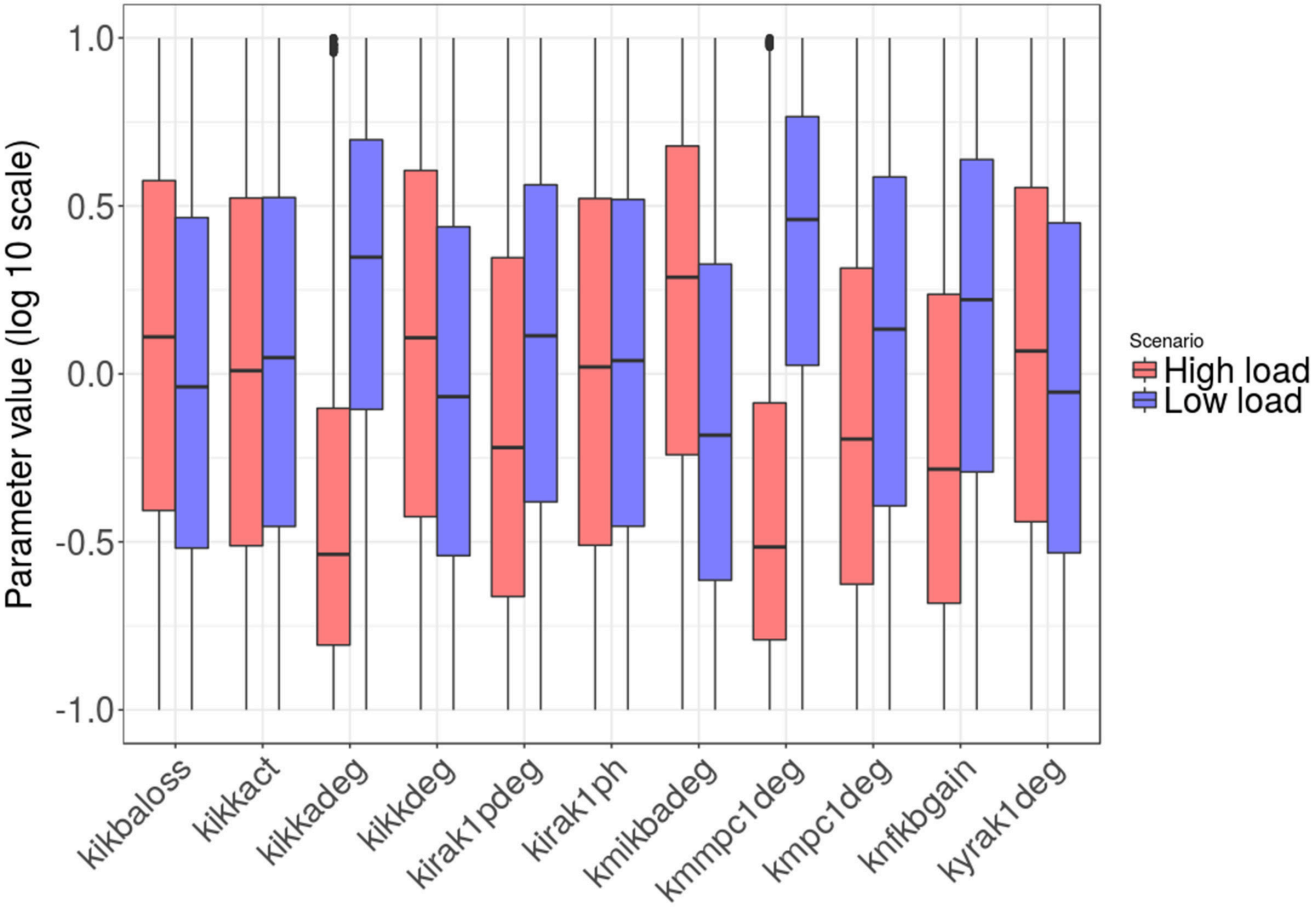
Distribution of the intracellular-level parameter values in the two groups: *high load* group in red and *low load* in blue. Only parameters with significant differences between groups are shown.

**Figure 9:**
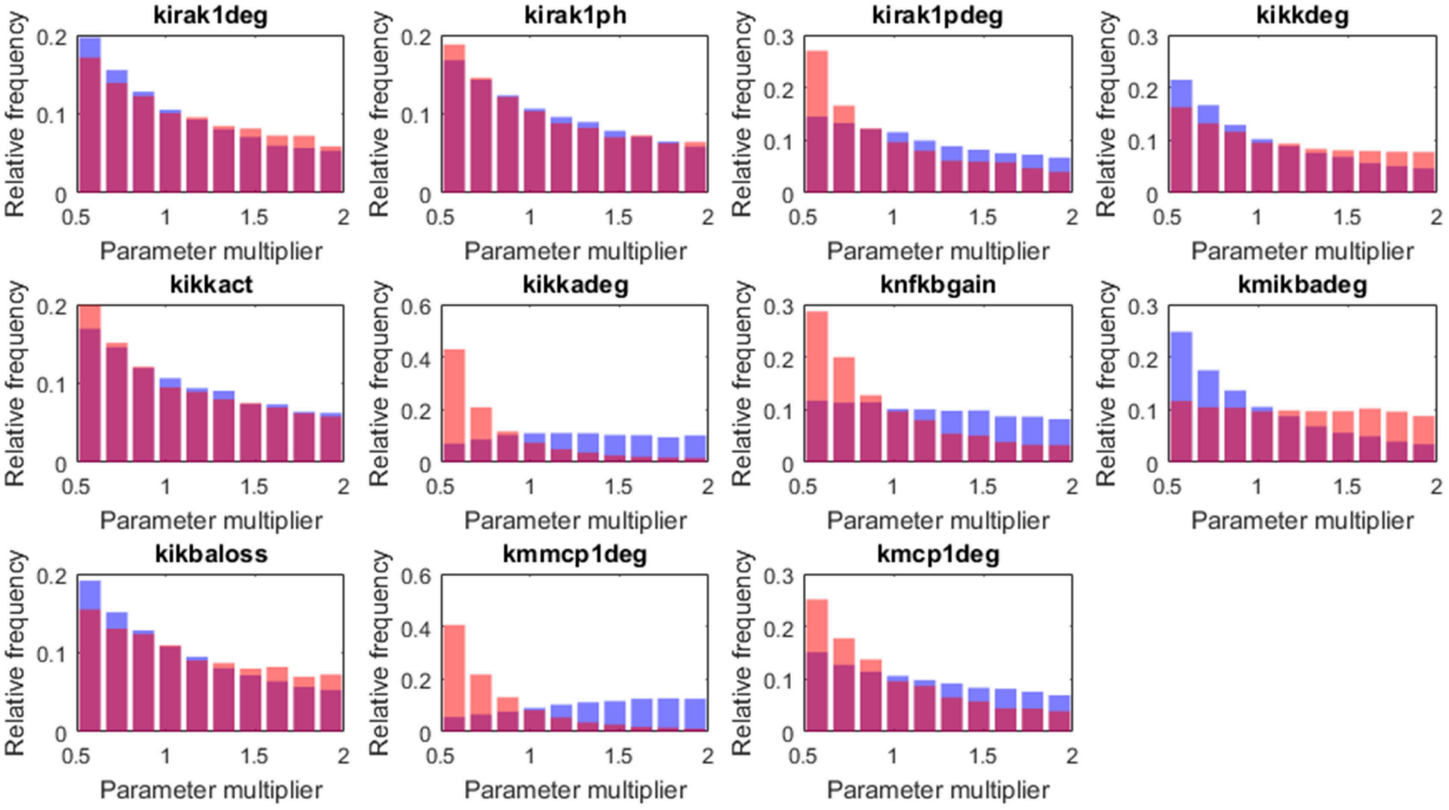
Histograms of the tissue level parameter values: *high load* group in red and *low load* in blue. We plotted the factors that multiply the value of the nominal solution. The distribution of solutions for a different sample number is shown in Supplementary Material.

Figures 8, 9 show the distribution of parameter values in both groups of solutions. According to the logistic regression analysis and Figure 8, the most relevant parameters distinguishing *high load* from *low load* are the degradation rate of the MCP-1 mRNA (*kmmcp1deg*), the degradation rate of the active form of IKK (*kikkadeg*), the degradation rate of the IκBa mRNA (*kmikbadeg*) and the production of NF-κB (*knfkbgain*). All these parameters together make for more than 70 % of the sum of the factors in the logistic regression (Table S5, Supplementary Material). The parameter *kmikbadeg* is higher in the *high load* group, and the other three are higher in the *low load* group.

### Decision tree analysis

When we applied the logistic regression to analysis of the populations of solutions, the solutions were divided into only *two* groups, which reduced the resolution of the analysis. To obtain higher-resolution and more disjoint groups of solutions, we used a decision tree analysis. In this type of statistical model, multidimensional groups are separated into disjoint sets based on the sequential partition of the available solutions. In our case, the tree creates several groups of solutions distinguished by their average bacterial load, in such a way as to maximize the difference in average bacterial load inside each set while minimizing the number of sets. The leaves of the tree represent subpopulations with different values for the model parameters, which can be interpreted as different infection strategies employed by pneumococci.

**Figure 10:**
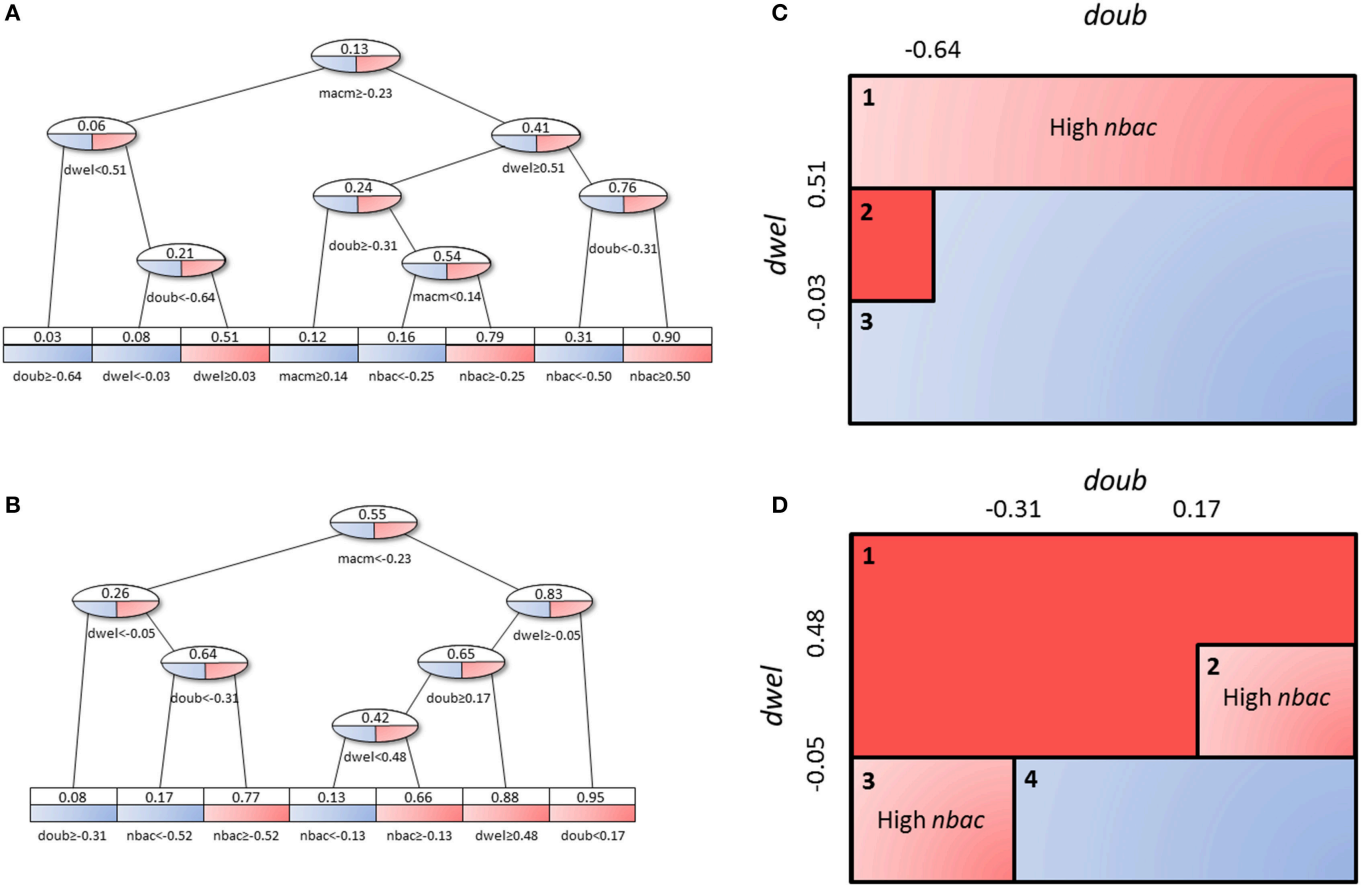
Decision tree analysis on tissue-level parameter values in the combined *low load* and *high load* groups. Red represents a set of solutions whose average infectivity is equal to or higher than 0.5, while blue stands for infectivity lower than 0.5. The intermediate steps (ovals) show both colors, as they cannot be classified unanimously at this level. Solutions with high macrophage movement speed **(A)** are separated from those with low macrophage movement speed **(B)**. The original tree can be seen in Figure S4. **(C,D)** represent an alternative depiction of the subpopulations from **(A,B)**, respectively. Three and four different subpopulations respectively were identified depending on the range of values of *dwel* and *doub*. Blue boxes indicate low-infectivity subgroups, dark red boxes high infectivity subgroups and light red boxes indicate subpopulations that achieve high infectivity if the criterion inside the box is met.

The decision tree analysis was performed at the tissue level in order to find subpopulations in the *high load* solutions which might account for different strategies employed by *S.p*. to infect the alveolar tissue. We defined infectivity as the continuous variable that fills the interval between the binary values of the *low load* and *high load* groups, where *high load* translates to a value of 1 and *low load* to one of 0. We denote “infection phenotype” as that with mean infectivity equal to or higher than 0.5, and “no infection phenotype” as that with mean infectivity lower than 0.5. The 20,027 solutions were analyzed using the *rpart* package in R. Figure 10 displays a representation of the results. In the leaves, the squared boxes at the bottom of the tree, an infectious phenotype is denoted by red coloring while blue indicates no infection. The intermediate branches are plotted in both colors if the decision tree cannot clearly separate the two outcomes.

Figures 10C,D shows a sketch of the subpopulations that can be inferred from Figures 10A, B, respectively. This sketch focuses only on three of the parameters (*doub, dwel*, and *nbac*). Figures 10A,C correspond to the half of the tree that segregates high macrophage velocity, while Figures 10B,D represent the other half.

First, in Figures 10A,C, we can find three subpopulations (1–3 in panel C). Subpopulation 1 represents bacteria with high dwelling probability, in which infectivity is high if the number of initial bacteria is high. Subpopulation 2 achieves very high infectivity and is made up of highly proliferative bacteria with intermediate dwelling probability. Subpopulation 3 combines low proliferation and low dwelling probability, and achieves low infectivity.

In Figures 10B,D, we observe four subpopulations. The first of these achieves high infectivity and is defined by intermediate to high proliferation and dwelling probability. If proliferation is low but dwelling probability is intermediate, as in subpopulation 2, high infectivity results only if the initial number of bacteria is high. Subpopulation 3 features the same constraint, i.e., dependence on the initial number of bacteria for high infectivity, but it relies on high proliferation and low dwelling probability. Finally, subpopulation 4 is characterized by low values of dwelling probability and low infectivity.

## Discussion

*Streptococcus pneumoniae* may prove a threat to life for immunocompromised patients, children and elderly people. An interesting strategy for the protection of these high-risk groups would be preventative interventions that stop the infection in its earliest stages, thus avoiding a systemic immune response; such interventions could be administered during periods of high infection risk (Cheng et al., 2015). Here, we propose harnessing the predictive ability of a mathematical model on the time course of infection in a single alveolus. The underlying idea is to make efficient use of modeling and simulations in order to conceive hypotheses on key physiological and bacterial parameters that distinguish between a successful infection and one rapidly cleared. Experiments can then be designed to test these hypotheses. Multi-level models allow for the integration of different temporal and spatial scales into a single mathematical model. This is particularly interesting in our context because alveolar infection involves interactions between the pathogen and the host cells at both the tissue and intracellular levels. In this work we have focused on the initial stages, an infection phase that involves essentially three types of cells (bacteria, epithelial cells, and macrophages) and a well-defined intracellular pathway.

Logistic regression analysis combined with systematic model perturbations and simulations provides a method for ranking model parameters and their associated processes in terms of their significance for the outcome of an early alveolar infection. The processes associated with the most important parameters could be investigated as targets for drug modulation in a preventative strategy.

At the tissue level, the most influential bacterial parameter predicted by the model and the logistic regression is the probability that the bacteria will remain in the same layer of the lining fluid. Transition between layers can be seen as a random process that alters the composition of the bacteria's capsule, thereby modifying adherence to the epithelial cells (Hammerschmidt et al., 2005); the higher the probability of this transition, the higher the turnover of the bacterial surface receptors that modify its adherence. Our model simulations suggest that a low rate of change to bacterial adherence to the epithelial cell surface is important for producing successful infection. Interestingly, this model prediction correlates with the function of a known *S.p*. virulence factor, the Pneumococcal surface protein C, which is involved in adherence to the epithelial surface (Iannelli et al., 2004).

It is important to highlight at this point that the model assumes a trade-off for the bacteria between adherence and clearance to the epithelial cell layer. Floating bacteria can be easily removed from the alveolus by the lining fluid. Conversely, attached bacteria can quickly be recognized by the epithelial cells, which will then produce MCP-1 to attract macrophages and promote clearance. Our model predicts that, during the very early stages of alveolar infection, the accumulation of a minimal biomass inside the alveolus by prevention of the lining fluid-driven removal of bacteria is critical to a productive infection. According to our simulations, this can be achieved by keeping bacteria attached to the epithelial cell layer. This choice would produce a more robust macrophage response through the MCP-1 produced by the epithelial cells. For the bacteria, fine tuning of the dwelling parameter can promote dispersion of the bacteria through the alveolus in such a way that some bacteria clusters could escape from macrophages without being washed out by the lining fluid.

In our analysis, increased proliferation of pneumococci promotes infection of the alveolar tissue. This suggests that faster growth can be beneficial for the establishment of infection. In previous studies, it has been observed that the capsule production efficacy can impose a delay in bacterial growth, and this effect influences virulence (Hathaway et al., 2012); in our model we observe a similar correlation via the effect of doubling time on infectivity. The number of initial bacteria entering the alveolus is another important parameter promoting infection. This is in line with the observation that nasopharyngeal colonization precedes alveolar infection. The experimental evidence in the literature suggests that, in order to achieve successful bacterial infection in the lung, pneumococci first need to accumulate in the higher respiratory tract, to then be released to the lower respiratory tissue (Mandell, 2009). Our simulations support this idea.

The capsule is a polysaccharide layer that protects bacteria from phagocytosis by macrophages. Bacteria colonizing the nasopharyngeal tissue produce low levels of capsule (Hammerschmidt et al., 2005). The model includes the hypothesis that, in order to invade the lung, bacteria trigger capsule production to protect themselves from macrophages. This hypothesis is supported by *in vitro* observations (AlonsoDeVelasco et al., 1995). In line with this, we considered in our model a dynamic ability of bacteria to produce capsule, triggered upon invasion of the alveolus. The results suggest that the adherence effect of the capsule could actually impair its protective effect during the initial phases of infection. This result may explain why some highly invasive strains of *S.p*. display lower efficiency in capsule production (Weinberger et al., 2009).

Resident macrophages are the first immunological barrier in the alveolar tissue; their task is to quickly clear invading pathogens and remove other particles without activating other branches of the innate and adaptive immune response. According to our analysis, two parameters accounting for the features of the alveolar macrophages play an important role in early infection: the number of resident macrophages and their speed of movement. Promisingly, these results provide an explanation for the higher susceptibility of infants to pneumococcal infections (O'Brien et al., 2009): in infants, alveolar macrophages tend to be insufficiently mature (Saito et al., 2008), and this lack of maturation impairs the movement of the resident macrophages through the alveolar lumen.

Moving to the intracellular level of the model, one of the most significant parameters in the analysis is related to the molecular stability of the MCP-1 mRNA (*kmmpc1deg*), which is consistent with some experimental observations (Rose et al., 2003). The analysis shows that high stability of the MCP-1 mRNA increases bacterial numbers at the end of the simulation. From the model (Figure 3), we can observe that local increases in the production of MCP-1 can act as chemokine traps in which macrophages are retained after they clear the bacteria cluster that triggered MCP-1 production. This arises from the fact that the rate of chemokine decay is much smaller than the velocity of macrophages (which can also be observed in time-lapse videos of the simulations available at http://sysbiomed-erlangen.weebly.com/resources.html).

A second influential parameter is the stability of IKK, an upstream kinase mediator in the NF-κB signaling pathway. Specifically, this kinase phosphorylates and thus marks for degradation IκB, a major inhibitor of NF-κB activation and nuclear shuttling (Jacobs and Harrison, 1998). Strikingly, our model predicts that high stability of the IKK protein increases the success rate of early alveolar infection (Figure 8). Long-term NF-κB activation provoked by a more stable IKK would increase the duration of MCP-1 release after a transient stimulation. This long-term signal would again trap the macrophages after clearance of bacteria, thereby delaying the elimination of bacteria at other sites.

The above two parameters were responsible for almost half of the variability of the regression model. If we add to them the production rate of NF-κB (*knfkbgain*) and the degradation rate of the mRNA of the NF-κB inhibitor IκBa (*kmikbadeg*), we see four parameters accounting for 70% of variability. The degradation of the IκBa mRNA is higher in the *high load* group, again increasing the duration of MCP-1 production because NF-κB cannot be re-sequestered efficiently if the stability of the IκBa mRNA is low. Finally, the production rate of NF-κB is lower in the *high load* group, a situation that would represent a low intracellular total amount of NF-κB. It has to be noted that this is the only parameter that refers to a production rate rather than a degradation rate. Our model simulations suggest that with a low total amount of intracellular NF-κB, the system is not able to trigger MCP-1 production and promote rapid, macrophage-mediated control of the infection.

Decision tree analysis is a methodology for finding subpopulations within defined groups. In the context of our analysis, we were looking for subpopulations of solutions within the *high load* and *low load* groups. We obtained five subpopulations with high infectivity and two with low infectivity (Figures 10A–D). Interestingly, the subpopulations differ depending on the macrophages' state of maturation. If the velocity of the macrophages is low we can differentiate four subpopulations, but if velocity is high, we can observe only three.

Further, the number and nature of subpopulations identified depend on the values of bacterial doubling time and adherence (*doub* and *dwel*, respectively). The final outcome of the subpopulations will also depend on the number of initial bacteria (*nbac*). We note that all three parameters relate to bacteria phenotypes. The decision tree analysis indicates that the strategies available to the bacteria depend on a single immunological parameter of the host, the velocity of their macrophages (*macm*). If the host is immunocompetent and has “quick” macrophages, the bacteria can only attain a highly infective phenotype in one of two ways: either by having a very high dwelling probability and a high initial number of colonizing bacteria, or by having very high proliferation with lower dwelling probability. Conversely, if the host macrophages move slowly, three out of the four identified bacterial phenotypes achieve high infectivity. In this case, a high number of bacteria can be reached for intermediate to high values of both proliferation and dwelling probability, with a reduced need for high initial numbers of colonizing bacteria. Analogously, if the initial number of bacteria is high, a strain with low proliferation and intermediate dwelling probability or with low dwelling probability and high proliferation is able to establish a productive infection. In our opinion, these subpopulations, distinguished by only three bacterial parameters (*dwel, doub*, and *nbac*), can explain observations of diversity in the infectivity of different *S.p*. serotypes (Serrano et al., 2006).

**Figure 11:**
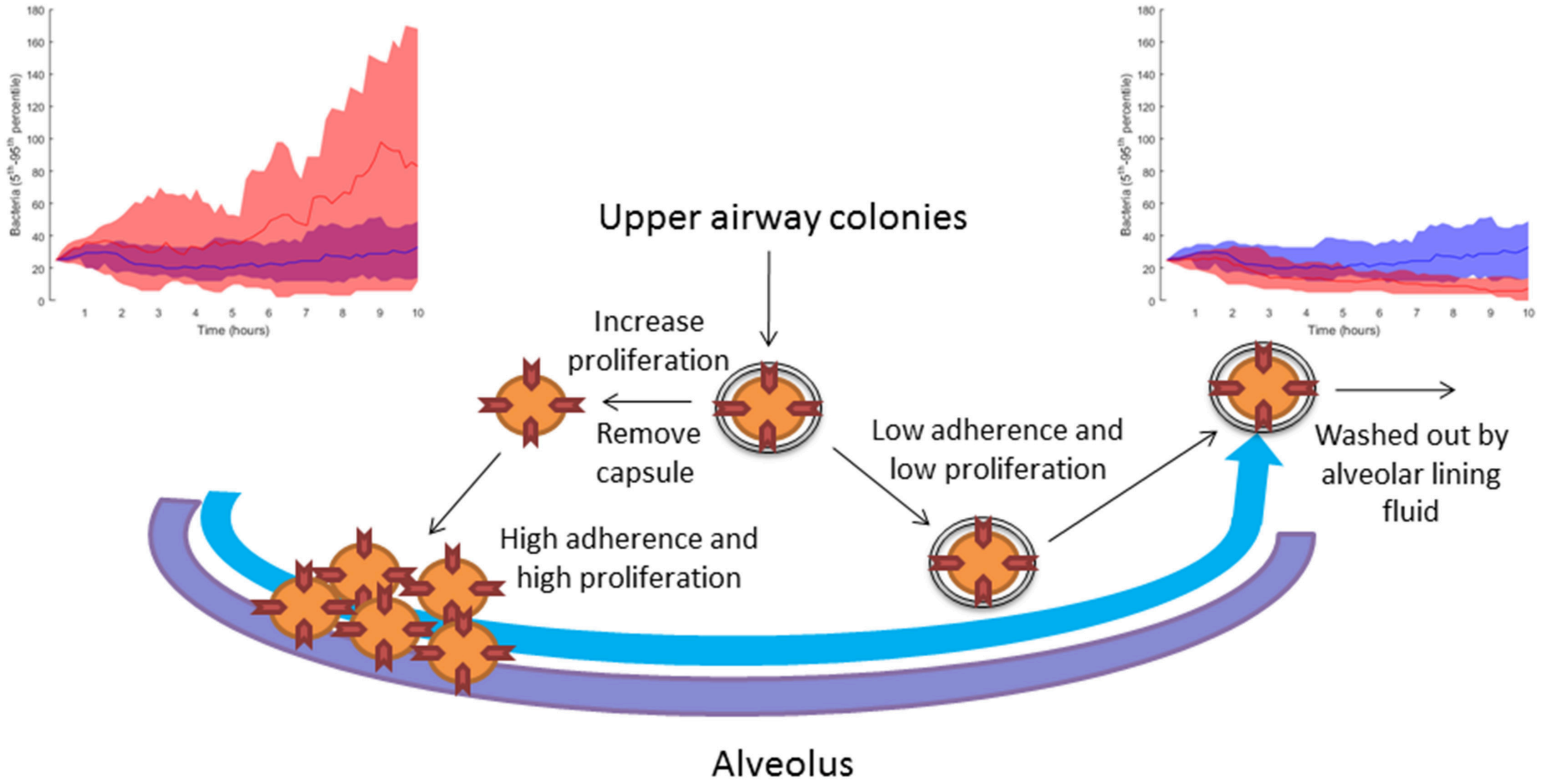
Sketch of the modeling-based predicted strategies pursued by bacteria attempting to infect the alveoli. When the bacteria increase their proliferation rate and decrease their capsule production, they can accumulate in the alveolus due to higher adherence to the epithelial tissue. The top left-hand diagram shows in red 10 simulations with reduced capsule production (*caps* = 5 h) and increased proliferation (*doub* = 100 min), compared to 10 simulations of the nominal solution in blue (see section Materials and Methods). If the bacteria keep their initial upper-airway phenotype of low proliferation and capsule production, they are washed out by the lining fluid and do not succeed in infecting the alveolus. The top right-hand diagram shows in red 10 simulations with increased capsule production (*caps* = 2 h) and reduced proliferation (*doub* = 400 min) compared to 10 simulations of the nominal solution in blue (see section Materials and Methods).

In Table 2, we link our model predictions to published experimental observations that support them, at least partially. For example, our model simulations suggest that dwelling probability and bacterial adherence play a key role in the establishment of infection. Specifically, the model predicts that adherence to epithelial cells facilitates bacterial infection. Since the capsule decreases the adherence of pneumococci, we hypothesize that the bacterial capsule is an obstacle during early infection (Figure 11). The invading bacteria originate from the upper airway colonies and are metabolically adapted to high capsule production and low proliferation (Hathaway et al., 2012). Our results indicate that, in order to succeed in infecting the alveolus, the bacteria have to switch to a higher proliferative profile and decrease capsule production to strengthen their adherence to the epithelial cells. In line with this, Iannelli et al. (2004) found in *S.p*. infection experiments with mice that bacterial adherence factors such as pneumococcal surface protein C can act as virulence factors, increasing the risk of severe infection and sepsis (Iannelli et al., 2004), although the authors did not succeed in fully unraveling the mechanism. Our model predictions provide a mechanistic explanation for Iannelli's results. In our view, our hypothesis can be tested in animal models using an antibody binding to one of the adherence factors of the bacteria, such as pneumococcal surface proteins. Preliminary studies have pointed to the utility of this approach (Ferreira et al., 2009).

**Table 2:** List of model predictions that are linked to published experimental results.

	**Experimental observation**	**References**
**INTUITIVE MODEL RESULT**		
Higher proliferation increases infectivity.	Higher capsule production efficacy correlates to virulence via faster growth.	Hathaway et al., 2012
Higher number of initial bacteria increases infectivity.	In order to reach a high number of invading bacteria in the lung, pneumococci first accumulate in the upper airways.	Mandell, 2009
Number and movement of macrophages affect infectivity.	Lower ability of infants to resist pneumococcal infection. Children lack mature alveolar macrophages.	Saito et al., 2008
Several bacterial subpopulations can be identified with different levels of infectivity.	Difference observed in invasion ability of S*treptococcus pneumoniae* serotypes. It has been observed that invasiveness is inversely correlated with frequency of commensal carriage.	Brueggemann et al., 2004
**COUNTER-INTUITIVE MODEL RESULT**		
Adherence to and higher dispersion through the epithelial surface increases infectivity.	Adherence protein Pneumococcal surface protein C is a virulence factor.	Iannelli et al., 2004
Capsule is an obstacle to infection.	Evidence of reduction of capsule production during first stages of invasion.	Hammerschmidt et al., 2005
The stability of the MCP-1 mRNA is higher in the *high load* group.	MCP-1 expression and protein production is increased in inflammatory lung diseases.	Wang et al., 2000

## Conclusions

This paper proposes a multilevel mathematical model to the end of unraveling pathogen-host interactions during the first 10 h of alveolar invasion. Combining simulations and techniques of statistical analysis, we identified various bacterial strategies for infection of the lower airways. These predicted strategies explain experimental differences observed in *S.p*. serotype infectivity. We propose that certain human pneumococci isolate phenotypes can be explained, at least in part, by differences in bacterial proliferation rates and adherence capabilities. On the basis of these findings, we hypothesize that interventions based on decreasing the adherence of pneumococci to alveolar epithelial cells would be able to protect high-risk populations before the disease is established.

## Author contributions

GS, XL, ME, and JV created the model; GS analyzed the model; GS, XL, ME, and JV discussed the results and wrote the manuscript.

### Conflict of interest statement

The authors declare that the research was conducted in the absence of any commercial or financial relationships that could be construed as a potential conflict of interest.
