# Supplementary material for: Bacterial Adherence and Dwelling Probability: Two Drivers of Early Alveolar Infection by Streptococcus pneumoniae Identified in Multi-Level Mathematical Modeling
Source: Front Cell Infect Microbiol. 2018 May 15;8:159. doi: 10.3389/fcimb.2018.00159 (PMC5962665; doi:10.3389/fcimb.2018.00159)
Supplement: Supplementary file 1 [file Data_Sheet_1.ZIP › Code/Readme.docx]

**Files**

Alveolus10bacs.avi: movie for the captions of the low bacteria solution (Figure 3A-D of the manuscript).

Alveolus100bacs.avi: movie for the captions of the high bacteria solution (Figure 3E-H of the manuscript).

AlveolusCapsuleAdh.m: modification of the code to include the adherence of the capsule.

AlveolusCapsuleAsyn.m: original code of the model.

AlveolusCapsulenoChem.m: modification of the code removing the chemoattractant signal of macrophages.

buscar_soluciones_cel_par.m: file to find random solutions for intracellular level parameters.

buscar_soluciones_tis_test.m: compare the simulations of the original code from the *noChem* code.

buscar_soluciones_tis_par.m: file to find random solutions for tissue level parameters.

CompareHypothesis.m: analyse the hypothesis of the chemokine attracting signal to macrophages.

epithelial.png: picture used by the model for the background.

Figures4_6_7_9.m: creates figures Figures 4, 6, 7 and 9 of the manuscript. Also creates the csv files and the files SolutionsMat.mat, Solutions2R.zip, SolutionsMatAdh.mat and Solutions2RAdh.zip.

Figures5_8LR&treesAR.zip: code in R to create figures 5 and 8, and to perform the logistic regression and the tree analysis using data from Solutions2R.zip and Solutions2RAdh.zip.

nameARcel.csv: names of cellular variables. Created in Figures4_6_7_9.m.

nameARcelAdh.csv: names of cellular variables, from the adherence modified code. Created in Figures4_6_7_9.m.

nameARtis.csv: names of tissular variables. Created in Figures4_6_7_9.m.

nameARtisAdh.csv: names of tissular variables, from the adherence modified code. Created in Figures4_6_7_9.m.

solsARcel.csv: solutions of cellular level. Created in Figures4_6_7_9.m.

solsARcelAdh.csv: solutions of cellular level, from the adherence modified code. Created in Figures4_6_7_9.m.

solsARtis.csv: solutions of tissular level. Created in Figures4_6_7_9.m.

solsARtisAdh.csv: solutions of tissular level, from the adherence modified code. Created in Figures4_6_7_9.m.

soluciones_Test1_10%.mat: solutions 10% below and above of the nominal solution from the original model.

soluciones_Test1_10%.mat: solutions 10% below and above of the nominal solution from the modified model without the chemokine signal.

Solutions2R.zip: all solutions merged in csv format, compressed in zip, from the adherence modified code. Created in Figures4_6_7_9.m.

Solutions2RAdh.zip: all solutions merged in csv format, compressed in zip. Created in Figures4_6_7_9.m.

SolutionsMat.mat: all solutions merged in matlab format. Created in Figures4_6_7_9.m.

SolutionsMatAdh.mat: all solutions merged in matlab format, from the adherence modified code. Created in Figures4_6_7_9.m.
