# Supplementary figures and images for: Bacterial Adherence and Dwelling Probability: Two Drivers of Early Alveolar Infection by Streptococcus pneumoniae Identified in Multi-Level Mathematical Modeling
Source: Front Cell Infect Microbiol. 2018 May 15;8:159. doi: 10.3389/fcimb.2018.00159 (PMC5962665; doi:10.3389/fcimb.2018.00159)

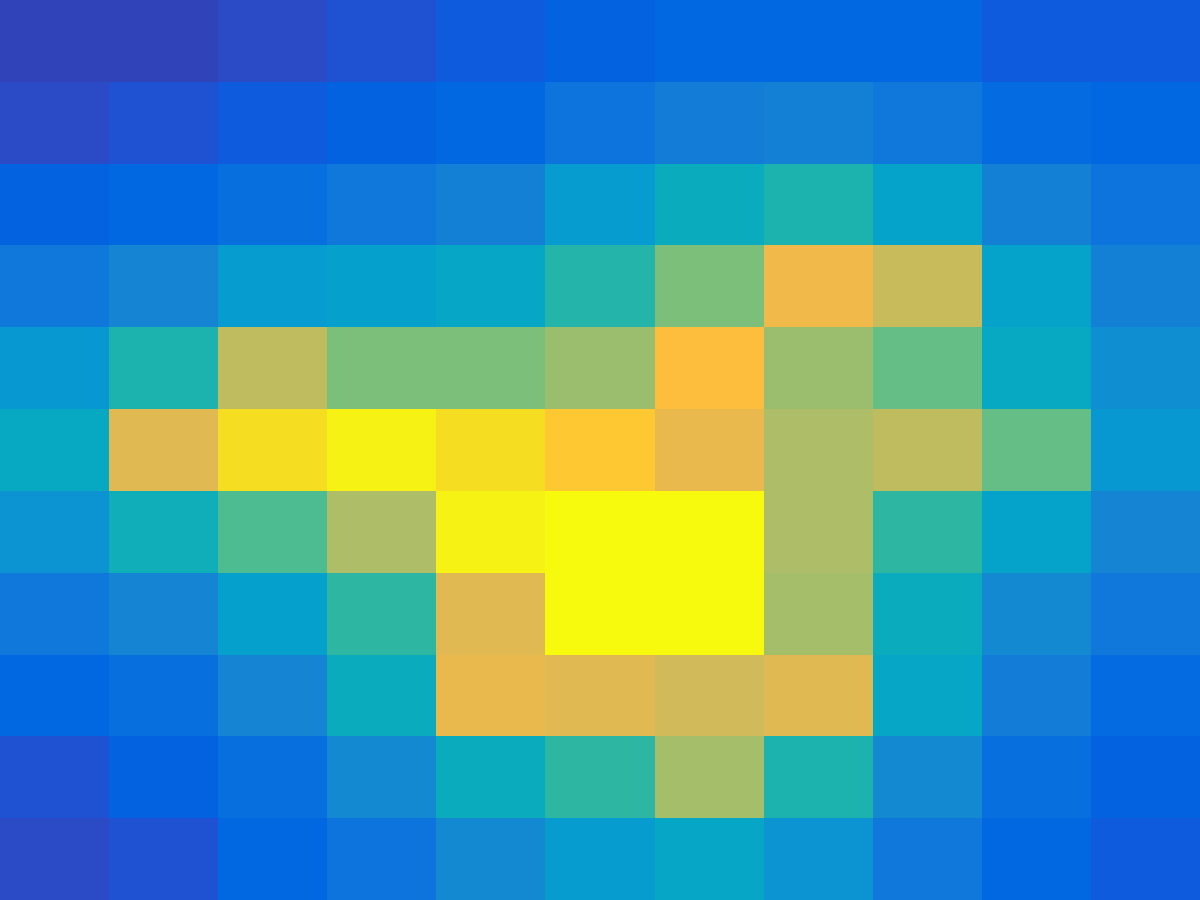

Supplement: Supplementary file 1 [file Data_Sheet_1.ZIP › Code/epithelial.png]
