# Supplementary material for: Bacterial Adherence and Dwelling Probability: Two Drivers of Early Alveolar Infection by Streptococcus pneumoniae Identified in Multi-Level Mathematical Modeling
Source: Front Cell Infect Microbiol. 2018 May 15;8:159. doi: 10.3389/fcimb.2018.00159 (PMC5962665; doi:10.3389/fcimb.2018.00159)
Supplement: Supplementary file 2 [file Data_Sheet_2.docx]

**Bacterial adherence and dwelling probability: two drivers of early alveolar infection by *Streptococcus pneumoniae* identified in multi-level mathematical modelling**

Guido Santos, Xin Lai, Martin Eberhardt and Julio Vera

Laboratory of Systems Tumor Immunology, Department of Dermatology, Universitätsklinikum Erlangen and Faculty of Medicine, Friedrich-Alexander University Erlangen-Nürnberg, Germany

## Intracellular signalling pathway

We have adapted the intracellular NF-κB network of the lung epithelial cells from a previous model developed in our group (Schulz et al., 2017). In so doing, we assumed that the model parameters for the intracellular network remain the same in the legionella-triggered system as in our current system with *S.p*. This assumption is supported by the fact that the NF-κB pathway is a central mediator of immune response to many different pathogens. We modified the receptor trigger kinetics, given that the receptors that recognise *S.p.* (TLR 4/2) differ from the one that recognises legionella (TLR5). As the kinetics of MCP-1 production and degradation are not well understood, we made the assumption that synthesis rates (*kmmcp1transc1*, *kmmcp1transc2* and *kmmcp1tranl*) are the same as for IL-8, while degradation rates (*kmmcp1deg* and *kmcp1deg*) were tuned in the parameter search due to their expected higher impact. In order to adapt the system to *S.p*., we used experimental data of lung epithelial cells stimulated *in vitro* with *S.p.* to calibrate the adapted model. Figure S5 shows the fit. Table S1 shows the intracellular parameter values and whether they were changed from the original model. We are confident that our nominal parametrisation represents an approximation of the physiological situation for two reasons: the assumption of conservation of the NF-κB pathway parameters is plausible, and the fit with the *S.p.* experimental data is good.


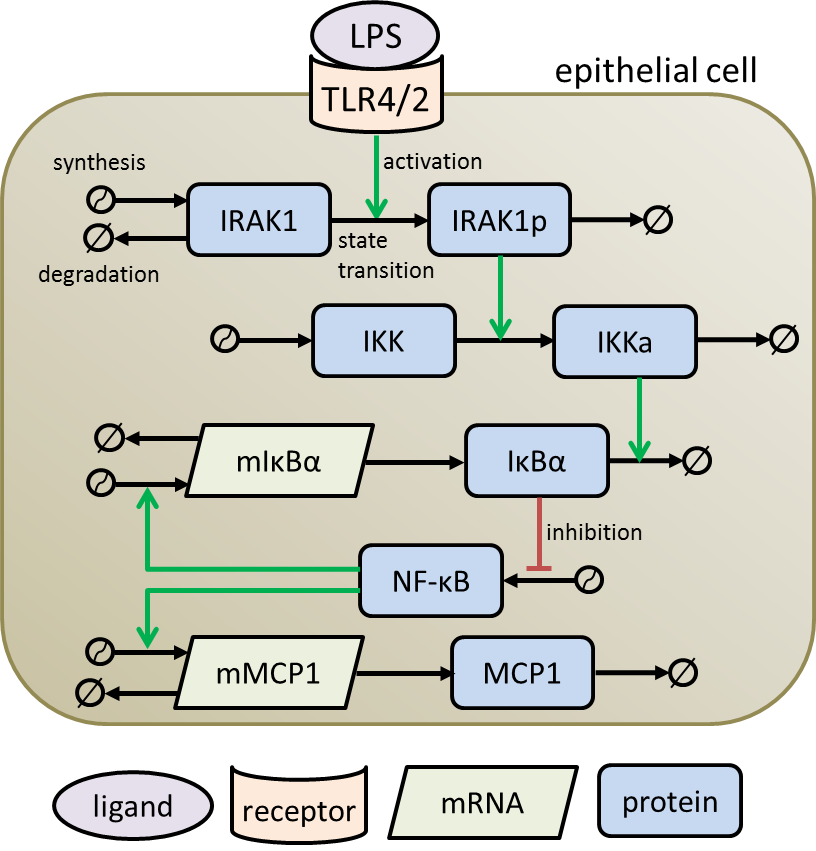

$$\frac{\boldsymbol{d}\left( \boldsymbol{IRAK}\boldsymbol{1} \right)}{\boldsymbol{dt}}\boldsymbol{=}\boldsymbol{k}_{\boldsymbol{IRAK}\boldsymbol{1}}^{\boldsymbol{syn}}\boldsymbol{-}\boldsymbol{k}_{\boldsymbol{IRAK}\boldsymbol{1}}^{\boldsymbol{deg}}\boldsymbol{\cdot IRAK}\boldsymbol{1-}\boldsymbol{k}_{\boldsymbol{IRAK1}}^{\boldsymbol{ph}}\boldsymbol{\cdot IRAK}\boldsymbol{1\cdot Bacteria (1)}$$

$$\frac{\boldsymbol{d}\left( \boldsymbol{IRAK}\boldsymbol{1}\boldsymbol{p} \right)}{\boldsymbol{dt}}\boldsymbol{=}\boldsymbol{k}_{\boldsymbol{IRAK}\boldsymbol{1}}^{\boldsymbol{ph}}\boldsymbol{\cdot IRAK}\boldsymbol{1\cdot Flagellin-}\boldsymbol{k}_{\boldsymbol{IRAK}\boldsymbol{1}\boldsymbol{p}}^{\boldsymbol{deg}}\boldsymbol{\cdot IRAK}\boldsymbol{1}\boldsymbol{p (2)}$$

$$\frac{\boldsymbol{d}\left( \boldsymbol{IKK} \right)}{\boldsymbol{dt}}\boldsymbol{=}\boldsymbol{k}_{\boldsymbol{IKK}}^{\boldsymbol{syn}}\boldsymbol{-}\boldsymbol{k}_{\boldsymbol{IKK}}^{\boldsymbol{act}}\boldsymbol{\cdot IKK\cdot IRAK}\boldsymbol{1}\boldsymbol{p-}\boldsymbol{k}_{\boldsymbol{IKK}}^{\boldsymbol{deg}}\boldsymbol{\cdot IKK (3)}$$

$$\frac{\boldsymbol{d}\left( \boldsymbol{IKKa} \right)}{\boldsymbol{dt}}\boldsymbol{=}\boldsymbol{k}_{\boldsymbol{IKK}}^{\boldsymbol{act}}\boldsymbol{\cdot IKK\cdot IRAK}\boldsymbol{1}\boldsymbol{p-}\boldsymbol{k}_{\boldsymbol{IKKa}}^{\boldsymbol{deg}}\boldsymbol{\cdot IKKa (4)}$$

$$\frac{\boldsymbol{d}\left( \boldsymbol{NFkB} \right)}{\boldsymbol{dt}}\boldsymbol{=}\boldsymbol{k}_{\boldsymbol{NFkB}}^{\boldsymbol{gain}}\boldsymbol{\cdot IKKa\cdot}\frac{\boldsymbol{N}_{\boldsymbol{tot}}\boldsymbol{-NFkB}}{\boldsymbol{K}_{\boldsymbol{1}}\boldsymbol{+IkB\alpha+IKKa}}\boldsymbol{-}\boldsymbol{k}_{\boldsymbol{NFkB}}^{\boldsymbol{loss}}\boldsymbol{\cdot}\frac{\boldsymbol{IkB\alpha\cdot NFkB}}{\boldsymbol{K}_{\boldsymbol{2}}\boldsymbol{+NFkB}}\boldsymbol{(5)}$$

$$\frac{\boldsymbol{d}\left( \boldsymbol{mIkB\alpha} \right)}{\boldsymbol{dt}}\boldsymbol{=}\boldsymbol{k}_{\boldsymbol{mIkB\alpha}}^{\boldsymbol{transc}}\boldsymbol{\cdot NFkB-}\boldsymbol{k}_{\boldsymbol{mIkB\alpha}}^{\boldsymbol{deg}}\boldsymbol{\cdot mIkB\alpha}\boldsymbol{(6)}$$

$$\frac{\boldsymbol{d}\left( \boldsymbol{IkB\alpha} \right)}{\boldsymbol{dt}}\boldsymbol{=}\boldsymbol{k}_{\boldsymbol{IkB\alpha}}^{\boldsymbol{transl}}\boldsymbol{\cdot mIkB\alpha-}\boldsymbol{k}_{\boldsymbol{IkB\alpha}}^{\boldsymbol{loss}}\boldsymbol{\cdot IKKa\cdot IkB\alpha\cdot}\frac{\boldsymbol{(N}_{\boldsymbol{tot}}\boldsymbol{-NFkB)}}{\boldsymbol{K}_{\boldsymbol{3}}\boldsymbol{+IkB\alpha+IKKa}}\boldsymbol{(7)}$$

$$\frac{\boldsymbol{d}\left( \boldsymbol{mMCP}\boldsymbol{1} \right)}{\boldsymbol{dt}}\boldsymbol{=}\boldsymbol{k}_{\boldsymbol{mMCP}\boldsymbol{1}}^{\boldsymbol{transc}\boldsymbol{1}}\boldsymbol{+}\boldsymbol{k}_{\boldsymbol{mMCP}\boldsymbol{1}}^{\boldsymbol{transc}\boldsymbol{2}}\boldsymbol{\cdot NFkB-}\boldsymbol{k}_{\boldsymbol{mMCP1}}^{\boldsymbol{deg}}\boldsymbol{\cdot mMCP}\boldsymbol{1}\boldsymbol{(8)}$$

$\frac{\boldsymbol{d}\left( \boldsymbol{MCP}\boldsymbol{1} \right)}{\boldsymbol{dt}}\boldsymbol{=}\boldsymbol{k}_{\boldsymbol{MCP}\boldsymbol{1}}^{\boldsymbol{transl}}\boldsymbol{\cdot mMCP}\boldsymbol{1-}\boldsymbol{k}_{\boldsymbol{MCP}\boldsymbol{1}}^{\boldsymbol{deg}}\boldsymbol{\cdot MCP}\boldsymbol{1(9)}$

Figure S1. Modelling scheme of the intracellular NF-κB pathway in lung epithelial cells. Upon receptor activation by bacterial LPS, IRAK1 is phosphorylated and the phosphorylated IRAK1 subsequently activates IKK. The activated IKK promotes the degradation of IκBα to release NF-κB. Free NF-κB enters the nucleus and promotes the production of its negative regulator IκBα and the chemokine MCP-1 through transcriptional activation. TLR4/2, toll-like receptor 4 or 2; IRAK1, IL-1R-associated kinase; IKK, IκB kinase; NF-κB, nuclear factor kappa-light-chain-enhancer of activated B cells; IκBα, NF-κB inhibitor alpha; MCP-1, monocyte chemoattractant protein 1. The characterisation of model parameters and the analysis of parameter identifiability are described in Schulz et al. (2017).

B

A


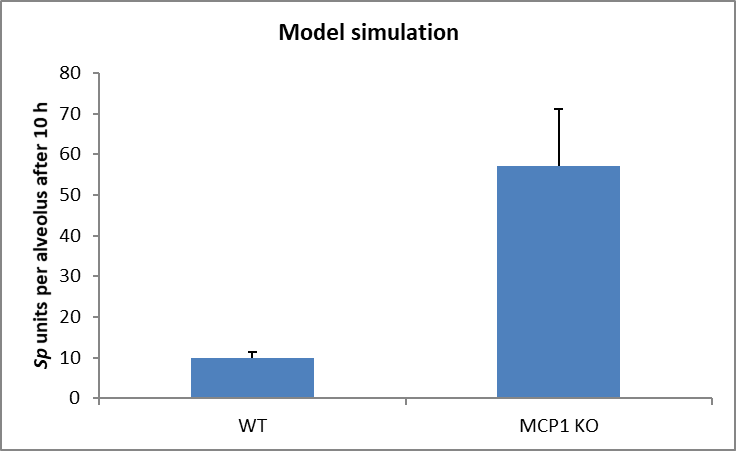

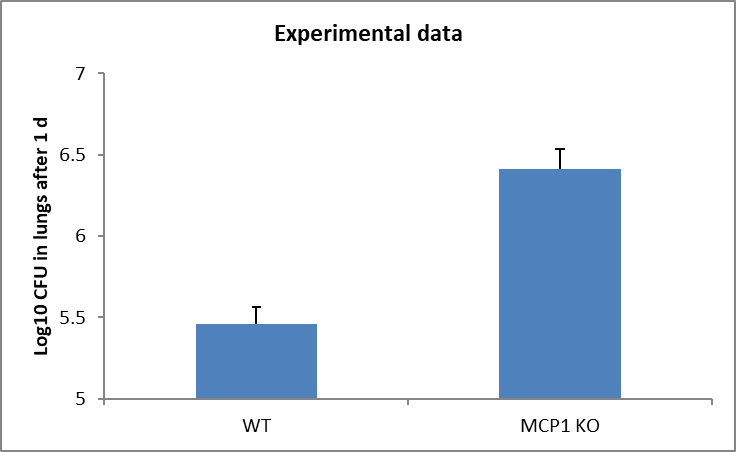


Figure S2. Effect of removing the MCP1 on the S.p. bacteraemia. A. Experimental measurement (mean ± SEM, n = 10) of the number of S.p. CFU in the whole lung of WT and MCP1 KO mice 1 day after infection, taken from (8). B. Simulation of the number of S.p. (mean ± SEM, n = 50) in a single alveolus with and without production of MCP1 10 hours after infection.


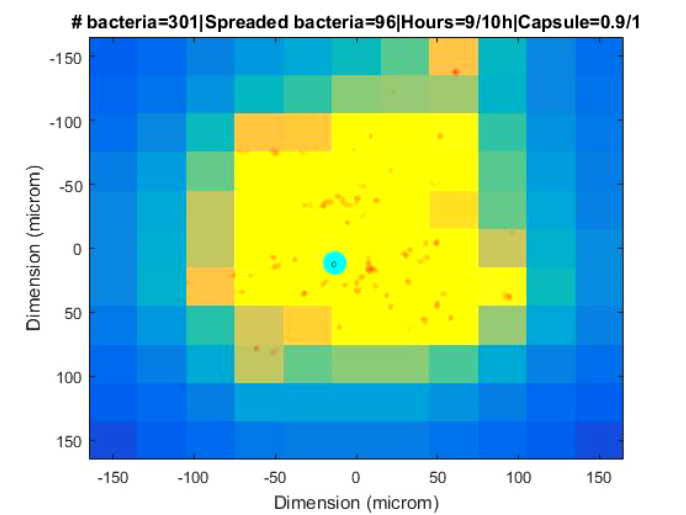


Figure S3. Panel H of Figure 3 of the main article. After 10 hours, the bacteria have spread through a wide area on the alveolus. The macrophage is not fast enough to clear all of them and the infection has become productive.


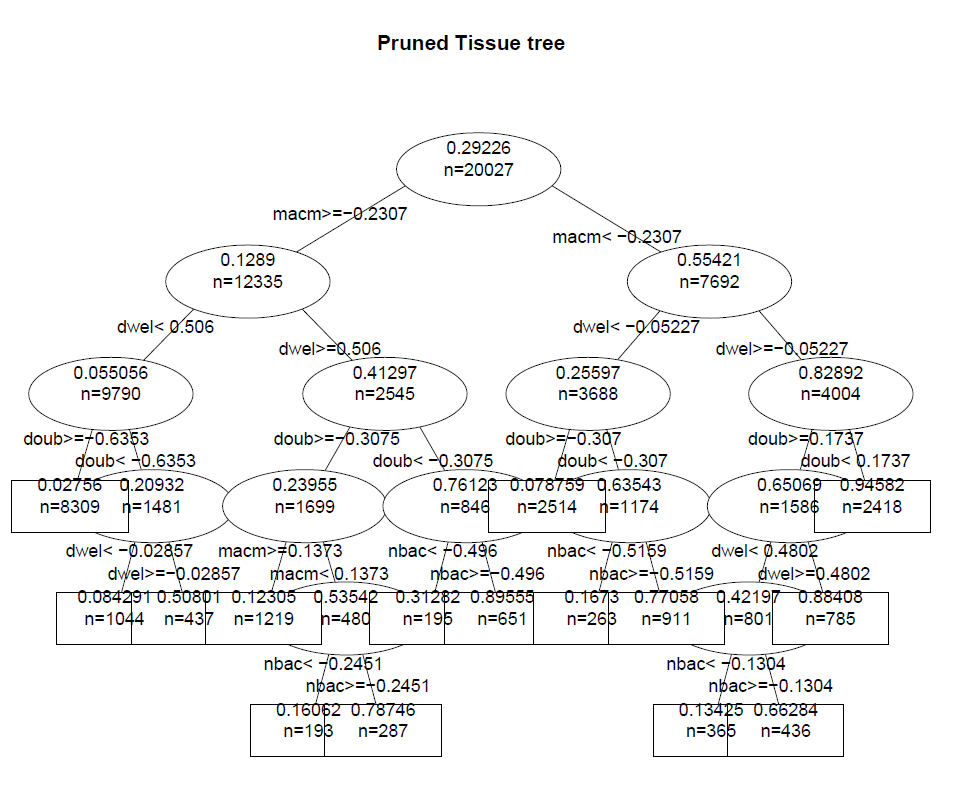


Figure S4. Histograms of the tissue-level parameter values: *high bacteria* group in red and *low bacteria* in blue. The values represented are the factors that multiply the value of the nominal solution. Three sets of solutions are displayed simultaneously to enable observation of the effect of the sample sizes (21557, 2180, or 233) on the distribution.

Tissue-level scale of model

Bacteria divide every 200 minutes (doub parameter; this value corresponds to the nominal solution), but each bacterium has its own internal time counter. The initial bacteria (nbac) are defined together with a random number between 0 and 200, indicating the time elapsed since the last division of each bacterium. When one bacterium has reached 200 minutes in its internal counter, it will generate a copy of itself in its current position. Bacteria have another attribute, the state in which they are present in the alveolus, which can be either attached to the alveolar epithelial cell layer or floating in the lining fluid. Both attached and floating bacteria will follow a random walk through the alveolus; the difference between them is the size of the step during the random walk, which is higher in the floating stage (flom) than in the attached stage (srfm). Bacteria can change between stages at any time iteration (1 minute); the probability of not changing in the next step is defined by the parameter dwel. When bacteria are in the floating stage, they are moved by the flow of lining fluid (radm) that pushes them out of the alveolus (from the centre to the border of the landscape). When they are attached, they are not moved by the lining fluid flow, but they can be recognised by the epithelial cells and trigger an intracellular signalling process to produce the MCP-1 chemokine to attract macrophages. The intensity of the signal that bacteria trigger to the epithelial cell is proportional to the amount of bacterial antigens presented to the epithelial cell during the contact (antg). Finally, bacteria produce a capsule, a process which commences at the beginning of the simulation. This is a dynamic process followed by a saturation kinetics (time/(time+caps)); the amount of capsule is determined by the previous expression and is confined between 0 and 1.

The initial number of alveolar macrophages is defined by the parameter nmac. The macrophages follow a random walk movement through the alveolar inner surface, with a step size defined in macm. Macrophages on top of one epithelial cell can feel the chemokine (MCP-1) concentration on top of the four closest epithelial cells. If the concentration of MCP-1 is higher in the epithelial cell in which the macrophage is located than in the four others, or equal to it, it will move randomly in the direction of any of the four closest epithelial cells. If this is not the case, the macrophage will take the next step in the direction of the epithelial cell in which the concentration of MCP-1 is highest. When one macrophage is at a distance to a bacterium lower than the distance defined by lpss, then the macrophage will always move towards the location of this bacterium. When one macrophage “touches” one bacterium, the bacterium is counted as “eaten” and disappears from the landscape. Beginning from the point in time when one bacterium is touched, an internal counter of the number of bacteria eaten inside the macrophage will increase by one and then this count will start to decrease through digestion. This decrease of the ingested bacteria follows an exponential decay (bacteria·exp(-phag·time)). The parameter of this exponential function is phag. If the count of bacteria inside one macrophage goes above the value defined by the parameter maxb, then the macrophage dies and disappears from the landscape. Finally, the MCP-1 chemokine diffuses through the alveolar surface following Fick’s Law, with a diffusion constant defined by the difk parameter. All the parameters presented here can be found in Table S1.

Tissue and cellular scales: merging and modelling

In order to merge the two different scales of the mathematical model (tissue and cellular scale), we performed an alternating strategy as follows: We start by running the tissue-level module of the model during the first 10 minutes of the simulation. This time step is robust enough to not produce substantial differences if we increase the size. Figure S8 shows the correlation of simulations between a 10-minute and 5-minute time step. Further, the experimental data in Schultz et al. 2017 (Schulz et al., 2017) used to construct several elements of our model indicate that the secretion of cytokines and chemokines after stimulation of lung epithelial cells takes several hours. Given that the secretion of these factors is the connection between the intracellular and the tissue levels of the model, we have assumed that a time interval of 10 minutes between each update of the models is acceptable. During this period, some of the initial bacteria that are defined as floating will become attached to the epithelial layer. After these first 10 minutes, the intracellular model is run, taking as the initial condition the last stage of the tissue-level model. The epithelial cells that have at least one bacterium on top of them would trigger an intracellular signal with a magnitude proportional to the number of bacteria on top of this cell. The remaining cells will not produce any signal. In order to establish how much MCP-1 will be produced by these cells, the intracellular model is run for another 10 minutes, during which the MCP-1 is accumulated outside each cell and starts diffusing through the alveolus. The last distribution of the MCP-1 concentration is taken as the initial condition for the next step of the tissue-level simulation. The macrophages will start following the gradient of this stage during the next 10 minutes. Additionally, the bacteria will move and (eventually) proliferate. This will produce another initial condition for the intracellular module, and the entire process is repeated. This stepwise series simulation of both scales is repeated until the end of the simulation (10 hours).


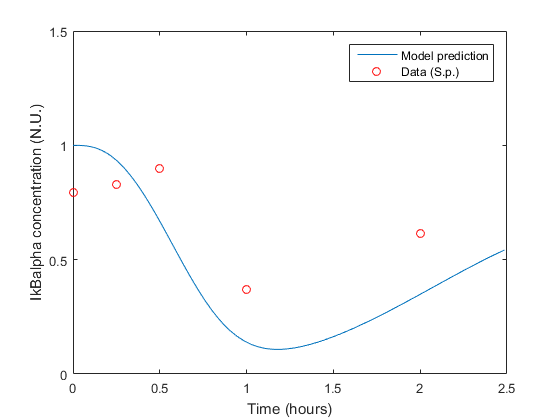


Figure S5. Manual calibration of the mathematical model of IκBα dynamics to the experimental data from (N’Guessan et al., 2006). The fitting strategy was chosen to bring the mathematical model in Figure S1 into line with the qualitative dynamics shown in the data. The most important feature of the data is reduction in the IκBα concentration followed by a slow recovery. This temporary reduction is responsible for the brief activation of NF-κB. We reproduced this pattern with our model by adjusting the free parameters in Table S2.


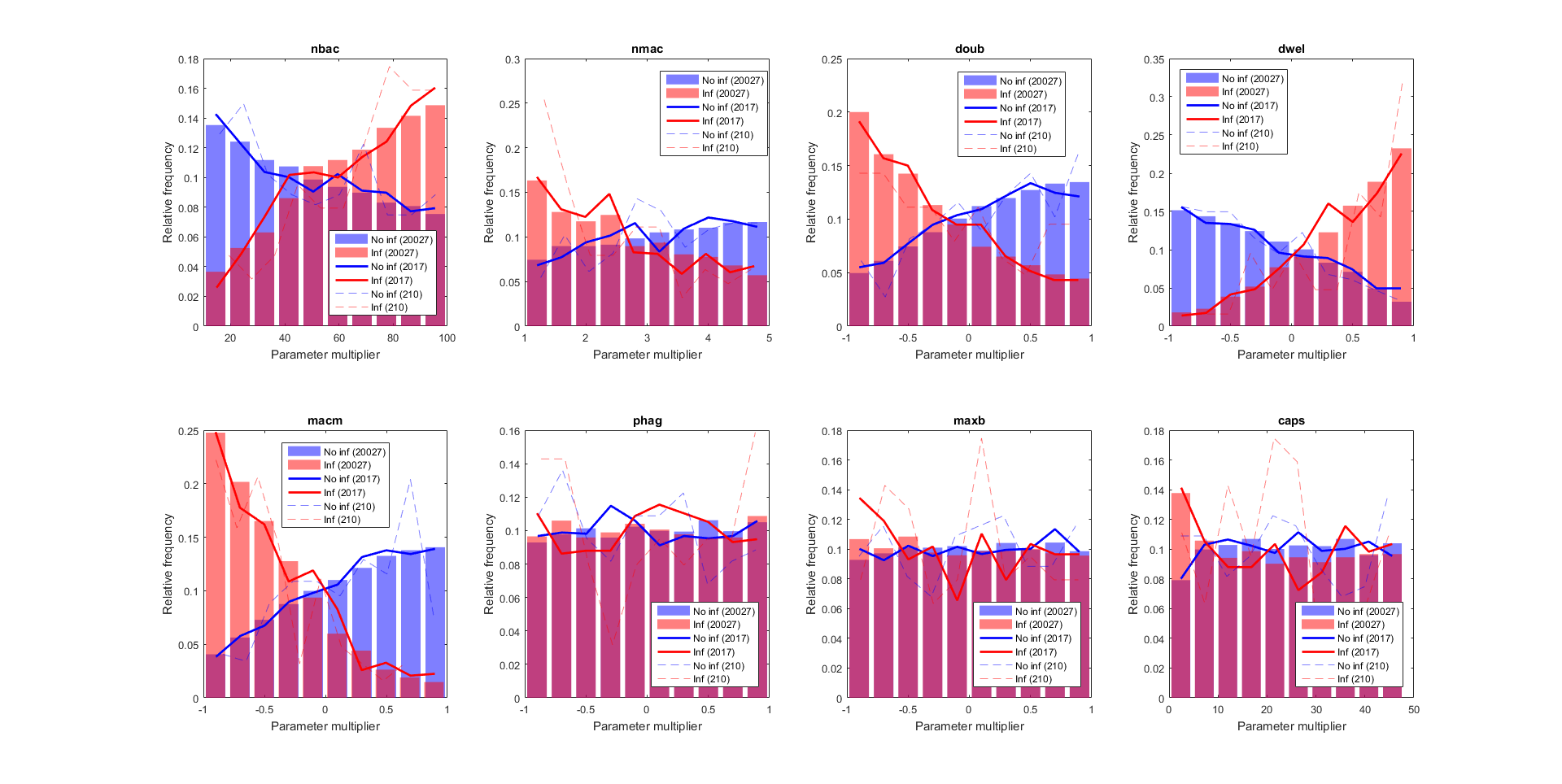


Figure S6. Histograms of the intracellular-level parameter values: *high load* group in red and *low load* in blue. The plotted values are the factors that multiply the value of the nominal solution. Three sets of randomly chosen solutions are displayed simultaneously to demonstrate the impact of the sample size on the distribution.


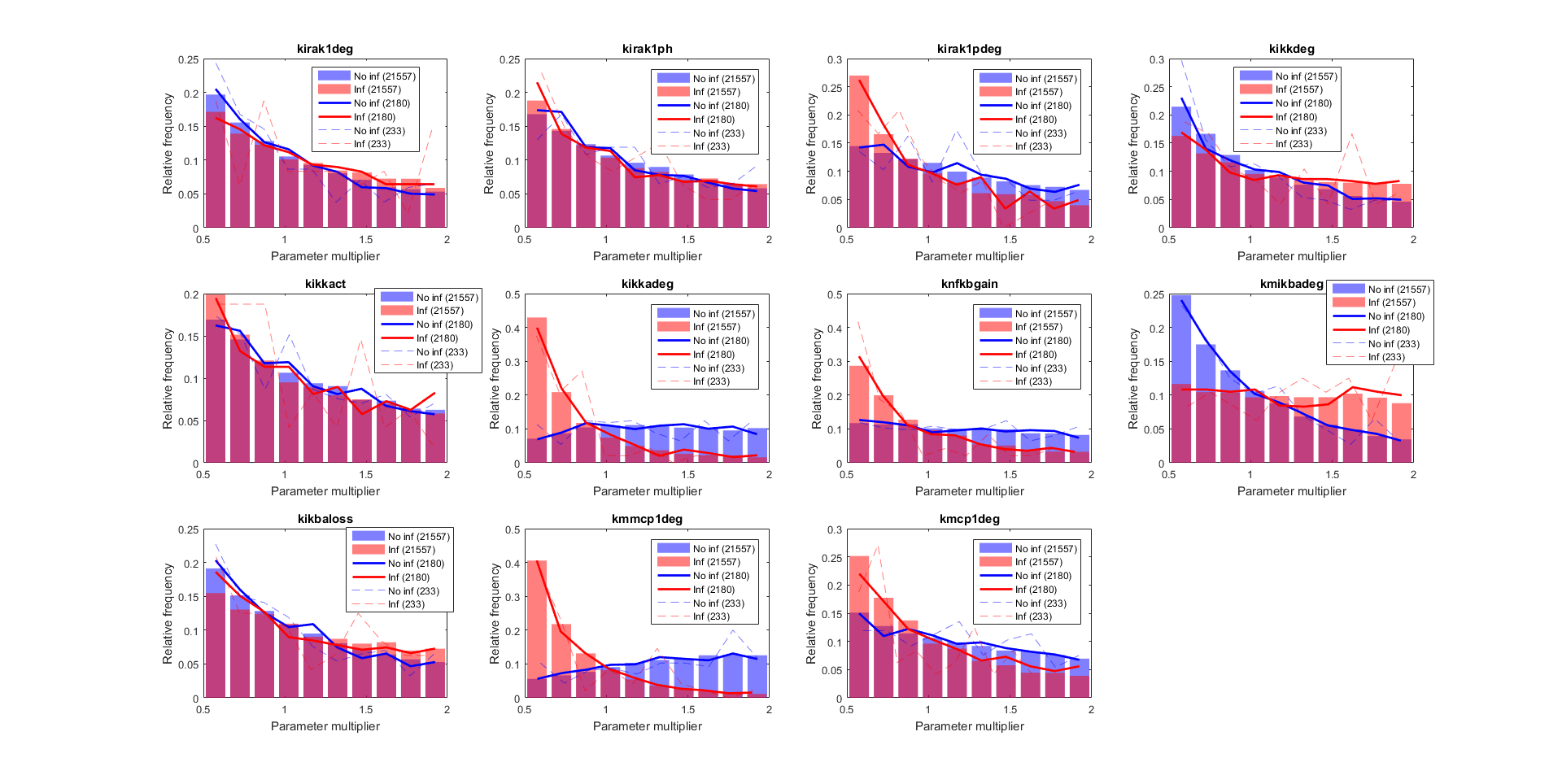


Figure S7. Histograms of the cellular-level parameter values: *high bacteria* group in red and *low bacteria* in blue. The values represented are the factors that multiply the value of the nominal solution. Three sets of solutions are displayed simultaneously to illustrate the effect of the sample sizes (21557, 2180, or 233) on the distribution.


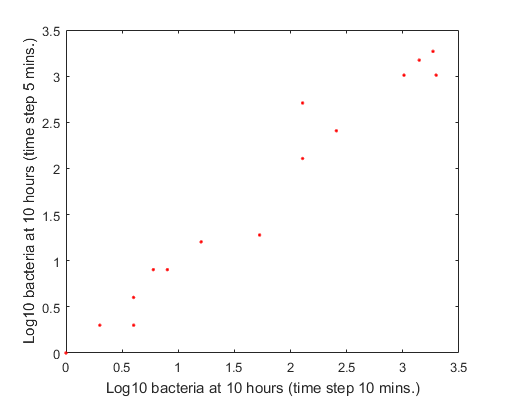


Figure S8. Correlation between numbers of bacteria at 10 hours, using different time steps in order to merge the tissue and cellular level models.

Table S1. Nominal parameter values for the tissue level.

| Name | Value | Reference |
| --- | --- | --- |
| Number of bacteria (nbac) | 1 bac | Arbitrary |
| Number of macrophages (nmac) | 1 mac | (Wallace et al., 1992), source: human |
| Chemokine diffusion coefficient (difk) | 6e4  min^-1^ | Assumed |
| Bacterial surface movement (srfm) | 0.1 μm/min. | Assumed |
| Bacterial floating movement (flom) | 3 μm/min. | Assumed |
| Fluid movement (radm) | 4.2e-2 μm/min. | (Lindert et al., 2007), source: mice |
| Bacterial doubling time (doub) | 200 min | (Ochs et al., 2004), source: human *in vitro* |
| Dwelling probability (dwel) | 0.999 n.u. | Estimated (constrained to the value that produces solutions for both *high load* and *low load* groups.) |
| Macrophage movement (macm) | 2 μm/min | (Khang, 2015), source: human *in vitro* |
| Phagocytosis rate (phag) | 0.048 min^-1^ | (Athamna and Ofek, 1988), source: human *in vitro* |
| Maximum bacteria per macrophage (maxb) | 50 bac | Estimated  (From the surface area ratio between a macrophage and a bacterium, it was calculated how many bacteria can be taken at once.) |
| Metabolic gradient (lpss) | 30 μm | Assumed |
| Time needed to build half of the capsule (caps) | 24 hours | 1-24 h range |

Table S2. Parameters overview of the model adapted from (Schulz et al., 2017).

| **Modified parameters** | **Description** | **Value for legionella** | **Updated value for *S.p.*** |
| --- | --- | --- | --- |
| $k_{IRAK}^{syn}$ | Synthesis rate of IRAK1 | 0.0961 h^-1^ | 0.0955 |
| $k_{IRAK}^{deg}$ | Degradation rate of IRAK1 | 0.0961 h^-1^ | 0.0955 |
| $k_{IL8}^{deg}/k_{MPC1}^{deg}$ | Degradation rate of IL8/MCP1 | 0.173 h^-1^ | 28.9 |
| **Unmodified parameters** | **Description** | **Value** | **Source** |
| $k_{IRAK1}^{ph}$ | Ligand-mediated phosphorylation rate of IRAK1 | 9.5x10-4 h^-1^ | Estimated |
| $k_{IRAK1p}^{deg}$ | Degradation rate of phosphorylated IRAK1 | 7.39 h^-1^* | Jensen and Whitehead, 2001 |
| $k_{IKK}^{syn}$ | Synthesis rate of IKK | 0.15 h^-1^ | Fixed |
| $k_{IKK}^{deg}$ | Degradation rate of IKK | 0.15 h^-1^ | Estimated |
| $k_{IKK}^{act}$ | Activation rate of IKK mediated by phosphorylated IRAK1 | 94 h^-1^ | Estimated |
| $k_{IKKa}^{deg}$ | Degradation rate of active IKK | 0.8 h^-1^ | Estimated |
| $k_{NFkB}^{gain}$ | Gain rate of free NFκB in the nucleus mediated by IκBα and IKKa | 4.9x10-3 h^-1^ | Estimated |
| $k_{NFkB}^{loss}$ | The loss of free NFκB mediated by IκBα | 1.8 h^-1^ | Estimated |
| $k_{mIkB\alpha}^{transc}$ | NFκB-mediated transcription rate of IκBα mRNA | 1.3x10^3^ h^-1^ | Estimated |
| $k_{mIkB\alpha}^{deg}$ | Degradation rate of IκBα mRNA | 1.6 h^-1^ | Estimated |
| $k_{IkB\alpha}^{transl}$ | Translational rate of IκBα from IκBα mRNA | 20 h^-1^ | Estimated |
| $k_{IkB\alpha}^{loss}$ | Loss of free IκBα mediated by NFκB and IKKa | 120 h^-1^ | Estimated |
| $k_{mMCP1}^{transc1}$ | Basal transcription rate of MCP1 mRNA | 4.9x10^-4^ h^-1^ | Estimated |
| $k_{mMCP1}^{transc2}$ | NFκB-mediated transcription rate of MCP1mRNA | 593 h^-1^ | Estimated |
| $k_{mMCP1}^{deg}$ | Degradation rate of MCP1 mRNA | 0.56 h^-1^ | Estimated |
| $k_{MCP1}^{transl}$ | Translation rate of MCP1 from its mRNA | 0.08 h^-1^ | Estimated |
| $K1, K2, K3$ | Michaelis–Menten coefficient | 1 n.u. | Fixed |
| $Ntot$ | Total amount of free NFκB | 1 n.u. | Fixed |

Table S3. Logistic regression results from glm (R 3.4.0) on the unmodified model at tissue level. The significance threshold was set at 0.001 (***).

| n = 20,027 | Estimate | Std. Error | z value | Pr(>\|z\|) |
| --- | --- | --- | --- | --- |
| nbac | 2.22571 | 0.05217 | 42.659 | <2e-16*** |
| nmac | -1.55792 | 0.04838 | -32.204 | <2e-16*** |
| doub | -3.26689 | 0.06276 | -52.053 | <2e-16*** |
| dwel | 4.31922 | 0.07360 | 58.681 | <2e-16*** |
| macm | -3.94172 | 0.06900 | -57.122 | <2e-16*** |
| phag | -0.10885 | 0.04329 | -2.514 | 0.0119 |
| maxb | -0.10294 | 0.04339 | -2.373 | 0.0177 |
| caps | -0.53919 | 0.04448 | -12.121 | <2e-16*** |

Table S4. Logistic regression results from glm (R 3.4.0) on the modified model at the intracellular level. In this version of the model, capsulated bacteria have decreased adherence: The higher the amount of capsule, the higher the probability of detachment from the epithelial cell layer. The significance threshold was set at 0.001 (***).

| n = 21,557 | Estimate | Std. Error | z value | Pr(>\|z\|) |
| --- | --- | --- | --- | --- |
| kyrak1deg | 0.25083 | 0.03873 | 6.476 | 9.39e-11*** |
| kirak1ph | -0.17614 | 0.03882 | -4.537 | 5.70e-06*** |
| kirak1pdeg | -0.70959 | 0.03954 | -17.947 | <2e-16*** |
| kikkdeg | 0.57153 | 0.03880 | 14.730 | <2e-16*** |
| kikkact | -0.20027 | 0.03856 | -5.194 | 2.06E-07*** |
| kikkadeg | -2.47295 | 0.04506 | -54.886 | <2e-16*** |
| knfkbgain | -1.33918 | 0.04048 | -33.085 | <2e-16*** |
| kmikbadeg | 1.22927 | 0.04033 | 30.482 | <2e-16*** |
| kikbaloss | 0.28976 | 0.03887 | 7.454 | 9.08e-14*** |
| kmmpc1deg | -2.87109 | 0.04537 | -63.281 | <2e-16*** |
| kmpc1deg | -0.85709 | 0.03932 | -21.795 | <2e-16*** |

Table S5. Logistic regression results from glm (R 3.4.0) on the modified model at tissue level (as in Table S4). In this regression, an additional term for the interaction between the capsule and the probability of transition was included. The significance threshold was set at 0.001 (***).

| n = 21,660 | Estimate | Std. Error | z value | Pr(>\|z\|) |
| --- | --- | --- | --- | --- |
| nbac | 1.91582 | 0.04551 | 42.099 | <2e-16*** |
| nmac | -1.35695 | 0.04299 | -31.563 | <2e-16*** |
| doub | -2.82088 | 0.05390 | -52.333 | <2e-16*** |
| dwel | 4.04702 | 0.06458 | 62.671 | <2e-16*** |
| macm | -3.49552 | 0.05908 | -59.164 | <2e-16*** |
| phag | -0.07949 | 0.03928 | -2.024 | 0.04301* |
| maxb | -0.12347 | 0.03959 | -3.119 | 0.00181** |
| caps | -0.23335 | 0.03979 | -5.864 | 4.52e-09*** |

Table S6. Logistic regression results from glm (R 3.4.0) on the unmodified model at the intracellular level. The significance threshold was set at 0.001 (***).

| n = 37,911 | Estimate | Std. Error | z value | Pr(>\|z\|) |
| --- | --- | --- | --- | --- |
| kyrak1deg | 0.30372 | 0.02834 | 10.719 | <2e-16*** |
| kirak1ph | -0.09453 | 0.02839 | -3.329 | 0.00087*** |
| kirak1pdeg | -0.74095 | 0.02894 | -25.599 | <2e-16*** |
| kikkdeg | 0.39397 | 0.02834 | 13.900 | <2e-16*** |
| kikkact | -0.13835 | 0.02829 | -4.891 | 1.00e-06*** |
| kikkadeg | -2.35420 | 0.03242 | -72.620 | <2e-16*** |
| knfkbgain | -1.29003 | 0.02965 | -43.513 | <2e-16*** |
| kmikbadeg | 1.15334 | 0.02929 | 39.380 | <2e-16*** |
| kikbaloss | 0.29548 | 0.02839 | 10.409 | <2e-16*** |
| kmmpc1deg | -2.74154 | 0.03279 | -83.616 | <2e-16*** |
| kmpc1deg | -0.78781 | 0.02863 | -27.521 | <2e-16*** |

**References**

Athamna, A., and Ofek, I. (1988). Enzyme-linked immunosorbent assay for quantitation of attachment and ingestion stages of bacterial phagocytosis. *J. Clin. Microbiol.* 26, 62–66.

Khang, D. (2015). Real time macrophage migration analysis and associated pro-inflammatory cytokine release on transparent carbon nanotube/polymer composite nano-film. *Nanotechnology* 26, 325101. doi:10.1088/0957-4484/26/32/325101.

Lindert, J., Perlman, C. E., Parthasarathi, K., and Bhattacharya, J. (2007). Chloride-dependent secretion of alveolar wall liquid determined by optical-sectioning microscopy. *Am. J. Respir. Cell Mol. Biol.* 36, 688–696. doi:10.1165/rcmb.2006-0347OC.

N’Guessan, P. D., Hippenstiel, S., Etouem, M. O., Zahlten, J., Beermann, W., Lindner, D., et al. (2006). Streptococcus pneumoniae induced p38 MAPK- and NF-kappaB-dependent COX-2 expression in human lung epithelium. *Am. J. Physiol. Lung Cell. Mol. Physiol.* 290, L1131-1138. doi:10.1152/ajplung.00383.2005.

Ochs, M., Nyengaard, J. R., Jung, A., Knudsen, L., Voigt, M., Wahlers, T., et al. (2004). The Number of Alveoli in the Human Lung. *Am. J. Respir. Crit. Care Med.* 169, 120–124. doi:10.1164/rccm.200308-1107OC.

Schulz, C., Lai, X., Bertrams, W., Jung, A. L., Sittka-Stark, A., Herkt, C. E., et al. (2017). THP-1-derived macrophages render lung epithelial cells hypo-responsive to Legionella pneumophila – a systems biology study. *Sci. Rep.* 7, 11988. doi:10.1038/s41598-017-12154-4.

Wallace, W. A., Gillooly, M., and Lamb, D. (1992). Intra-alveolar macrophage numbers in current smokers and non-smokers: a morphometric study of tissue sections. *Thorax* 47, 437–440.
